# Polyglutamate-based nanoconjugates for image-guided surgery and post-operative melanoma metastases prevention

**DOI:** 10.7150/thno.72941

**Published:** 2022-08-29

**Authors:** Yana Epshtein, Rachel Blau, Evgeni Pisarevsky, Shani Koshrovski-Michael, Dikla Ben-Shushan, Sabina Pozzi, Gal Shenbach-Koltin, Lidar Fridrich, Marina Buzhor, Adva Krivitsky, Pradip Dey, Ronit Satchi-Fainaro

**Affiliations:** 1 Department of Physiology and Pharmacology, Sackler Faculty of Medicine, Tel Aviv University, Tel Aviv 6997801, Israel.; 2 Department of NanoEngineering, University of California, San Diego, 9500 Gilman Drive, Mail Code 0448, La Jolla, CA 92093-0448

**Keywords:** Polymeric nanomedicine, Image-guided surgery, BRAF/MEK inhibitors, PGA, Immune checkpoint inhibitors

## Abstract

**Rationale:** Cutaneous melanoma is the most aggressive and deadliest of all skin malignancies. Complete primary tumor removal augmented by advanced imaging tools and effective post-operative treatment is critical in the prevention of tumor recurrence and future metastases formation.

**Methods:** To meet this challenge, we designed novel polymeric imaging and therapeutic systems, implemented in a two-step theranostic approach. Both are composed of the biocompatible and biodegradable poly(α,L-glutamic acid) (PGA) nanocarrier that facilitates extravasation-dependent tumor targeting delivery. The first system is a novel, fluorescent, Turn-ON diagnostic probe evaluated for the precise excision of the primary tumor during image-guided surgery (IGS). The fluorescence activation of the probe occurs via PGA degradation by tumor-overexpressed cathepsins that leads to the separation of closely-packed, quenched FRET pair. This results in the emission of a strong fluorescence signal enabling the delineation of the tumor boundaries. Second, therapeutic step is aimed to prevent metastases formation with minimal side effects and maximal efficacy. To that end, a targeted treatment containing a BRAF (Dabrafenib - mDBF)/MEK (Selumetinib - SLM) inhibitors combined on one polymeric platform (PGA-SLM-mDBF) was evaluated for its anti-metastatic, preventive activity in combination with immune checkpoint inhibitors (ICPi) αPD1 and αCTLA4.

**Results:** IGS in melanoma-bearing mice led to a high tumor-to-background ratio and reduced tumor recurrence in comparison with mice that underwent surgery under white light (23% *versus* 33%, respectively). Adjuvant therapy with PGA-SLM-mDBF combined with ICPi, was well-tolerated and resulted in prolonged survival and prevention of peritoneal and brain metastases formation in BRAF-mutated melanoma-bearing mice.

**Conclusions:** The results reveal the great clinical potential of our PGA-based nanosystems as a tool for holistic melanoma treatment management.

## Introduction

The primary treatment option for patients with early-stage melanoma is surgery 1. Advanced surgical technologies and new treatments prolong the life span of melanoma patients with primary tumors, however, metastatic spread may still occur later on. About 30% of melanoma patients who progress after primary tumor resection develop metastases in diverse sites such as liver, lungs, bones, and brain 2. Importantly, among all primary neoplasms in adults, melanoma has the highest inclination to metastasize to the brain 3. It was found that 75% of patients presented brain metastases at the time of death 4.

Improvements in surgical technologies seek to validate that no cancer cells remain at the incision of the surgical margins, *i.e.,* negative margins, during the tumor excision. Image-guided surgery (IGS) was developed to assist the surgeon by marking tumor margins in real-time with imaging agents that can be visualized by advanced imaging devices. Tremendous progress in this field is shown by numerous imaging and diagnostic Turn-ON probes that are optically silent (“OFF”) in their native form and are switched “ON” to emit fluorescence signal upon meeting certain enzymes or analytes at the site of interest. Several examples include fluorescently-tagged activatable cell-penetrating peptides (ACPP) 5, low molecular weight fluorogenic peptides, and trigger-activated analytes 6-10. Several imaging probes were assessed in clinical trials for their specificity, sensitivity and/or the accuracy of excising tumors during IGS 11-15, however, their ability to obtain negative tumor margins or lowering the incidence of disease recurrence is still unclear.

Nevertheless, local residual tumor micro-infiltration and circulating tumor cells that are under the limit of detection by current technologies continue to be the main cause for tumor recurrence and further metastatic colonization 16. Thus, there is a great motivation to improve surgery outcome by additional guidance of a smart probe, and then, to prevent the metastases formation by post-operative targeted treatment with minimal adverse effects. To that end, we set to develop an effective prophylactic treatment which consists of a combination of molecularly-targeted therapies and immunotherapy. More than 50% of all patients with malignant melanoma harbor an activating mutation in the BRAF kinase gene 17, which is a part of the mitogen-activated protein kinase (MAPK) signaling pathway involved in the regulation of cellular processes such as proliferation and survival 17, 18. Hence, new MAPK pathway-targeted therapeutics were developed. Examples for those that received FDA approval include BRAF inhibitors (BRAFi) vemurafenib 19, dabrafenib (DBF) 20, encorafenib 21 and MEK inhibitors (MEKi) trametinib (TRM), selumetinib (SLM) 22-24 and binimetinib 21.

Further paramount advances in melanoma treatment include recently emerged immune checkpoint inhibitors (ICPi) targeting checkpoint proteins, which are involved in suppressing the immune system. Among them, the monoclonal antibodies that target either the programmed cell death-1 (PD-1) receptor on infiltrating T cells, such as pembrolizumab 25 and nivolumab 26 or the cytotoxic T-lymphocyte antigen-4 (CTLA-4) such as ipilimumab 27. Combination therapy using αPD-1 and αCTLA-4 antibodies has been studied to enhance efficacy and reduce toxicity 28. Furthermore, recent evidence indicates a beneficial outcome for combining immunotherapies with BRAFi/MEKi 28. In addition to their tumor cell-intrinsic effects, BRAFi are reported to have immune cell-modulating effects, thus they may synergize with immune cells 29, 30. Inhibition of the MAPK pathway in both BRAF-mutant and wild type melanoma cells resulted in increased expression of melanocytes-differentiation antigens which is associated with improved recognition by antigen-specific T lymphocytes 31, 32. MAPK pathway-targeted therapies have resulted in significant clinical responses in a large proportion of melanoma patients, however, acquired resistance was frequently developed 33, as opposed to immunotherapies that have shown limited clinical responses, however, led to durable tumor regression and complete responses in a subset of patients 28. These potentially complementary treatment strategies led to numerous clinical trials evaluating the safety and efficacy of their combinations. Among them are the combinations of: Ipilimumab (αCTLA-4) with DBF/TRM evaluated for V600E/K mutation positive metastatic or unresectable melanoma patients (NCT01767454) in an ongoing phase I study 34; pembrolizumab (αPD-1 therapy) with DBF/TRM, in an ongoing phase I/II study, (NCT02130466) 35; and a phase III, advanced clinical trial testing atezolizumab (αPD-L1) with vemurafenib/cobimetinib as a first-line treatment in BRAF V600 mutation-positive patients with unresectable advanced melanoma (NCT02908672) 36. Several additional studies investigating various treatment combinations and regimens are ongoing, including Spartalizumab (PDR001; αPD1) and TRM/DBF (NCT02967692) 37, encorafenib/ binimetinib/ pembrolizumab (NCT02902042) 38, atezolizumab (αPD-L1) with vemurafenib/ cobimetinib (NCT02902029) 39, vemurafenib/ cobimetinib/ ipilimumab/ nivolumab (NCT02968303) 40. Surprisingly, preclinical studies examining the efficacy of MAPK/ERK inhibition with PD-1/CTLA-4 blockade in melanoma are lacking. A beneficial synergism of CTLA-4 blockade with BRAF inhibition was shown in BRAF-wildtype colon carcinoma and fibrosarcoma mouse models 41.

The benefits of macromolecules, particularly polymers exploited as vehicles for the delivery of low molecular weight (Mw) drugs and diagnostic agents were broadly described in terms of improved drugs solubility 42, simultaneous delivery of synergistic therapeutic combinations 43-45, or a large payload of a drug and/or imaging agent 46 to the tumor site. These led to enhancement of the fluorescence signal in comparison with low Mw fluorescent entities 47, 48 and altered the pharmacokinetics/pharmacodynamics (PK/PD) of drugs which present a narrow therapeutic window, hence increasing their maximum-tolerated dose (MTD) and decreasing their adverse effects thus improving their safety profile 49. We have recently reported the rational design and synthesis of a biocompatible and biodegradable poly(α,L-glutamic acid) (PGA) delivering an optimized combination of drugs targeting mutated BRAF and MEK in melanoma 43. The enhanced antitumor effect was demonstrated in primary melanoma-bearing mice treated with SLM and modified DBF (mDBF) conjugated to PGA (PGA-SLM-mDBF) compared to those treated with the combination of the unconjugated drugs or combination of the mono-drug conjugates 43. This was probably attributed to the macromolecular carrier, PGA, that simultaneously delivered the drugs in a synergistic ratio to the tumor, exploiting the enhanced permeability and retention (EPR) effect 50.

Taking together the high risk for tumor recurrence and brain metastases development when melanoma progresses, and the beneficial polymeric delivery of both diagnostics and combination of therapeutics to the tumor, we were motivated to design a two-step treatment approach based on our PGA backbone. For the IGS step, we designed a novel Turn-ON probe composed of PGA bearing Cy5 and Black Hole Quencher (BHQ) molecules, PGA-Cy5-BHQ (PCbhQ). The Cy5 and BHQ dense loading enables FRET interaction and optical quenching of the probe before its activation. Fluorescence activation of the probe was achieved by the degradation of the polymeric backbone in the presence of cysteine cathepsins that are overexpressed in various tumor types, including melanoma 43, 46. We explored this system in terms of quenching capacity, tumor cell internalization and Turn-ON abilities. Eventually, we examined the ability of PCbhQ to differentiate tumor boundaries from the healthy surroundings during the surgery and to prevent primary tumor recurrence. As the post-surgical treatment step, we proposed the combination of our PGA-SLM-mDBF with ICPi (αPD-1 and αCTLA-4). To the best of our knowledge, this is the first report of the combination of these ICPi with PGA bearing a synergistic ratio of BRAFi/MEKi for post-IGS prevention of metastases. We hypothesized that these targeted treatments, simultaneously delivered in a synergistic ratio, while combined with ICPi which further stimulate the host immunity will succeed to eliminate circulating or newly colonizing melanoma cells, thus preventing metastases development.

## Results

### Image-guided tumor resection as a first therapeutic step

#### PGA-Cy5-BHQ (PCbhQ) synthesis and characterization

**Figure 1:**
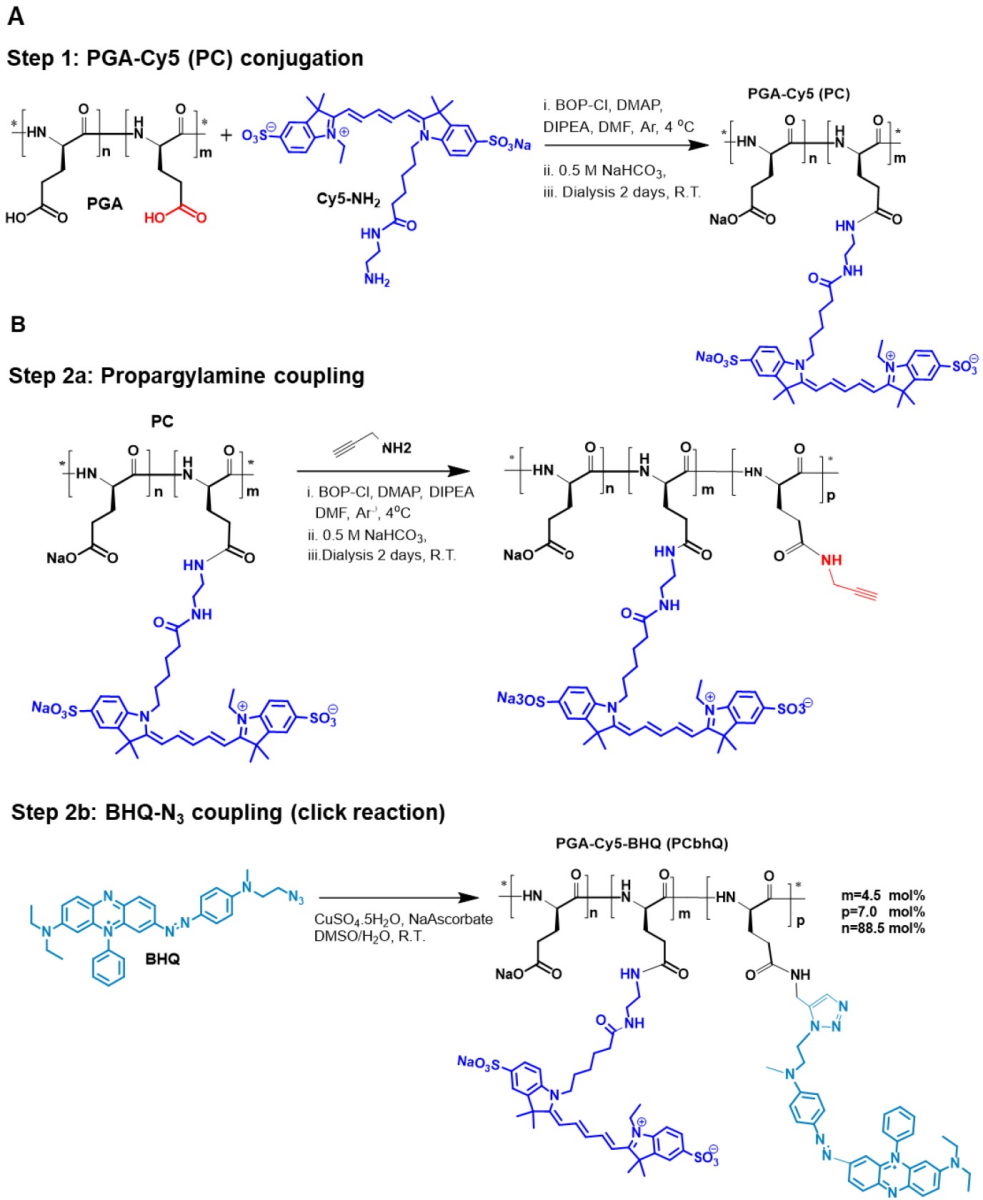
** Synthesis of PCbhQ Turn-ON probe. (A)** PGA-Cy5 (PC) synthesis and **(B)** subsequent conjugation of BHQ-N_3_ to create PCbhQ probe.

To create an efficiently quenched FRET-based PCbhQ Turn-ON probe, Cy5 fluorophores were conjugated to PGA backbone in combination with BHQ molecules (Figure 1). BHQ has a broad absorbance spectrum with λ_max_ = 672 nm, suitable for fluorescence signal attenuation of Cy5 (λ_max_ = 650 nm). The conjugation was performed in two steps (Figure 1A-B). The resulted PCbhQ conjugate had 4.5 mol% of Cy5 and 7 mol% of BHQ, determined optically and confirmed by ^1^H NMR analysis (Figure S1A).

**Figure 2:**
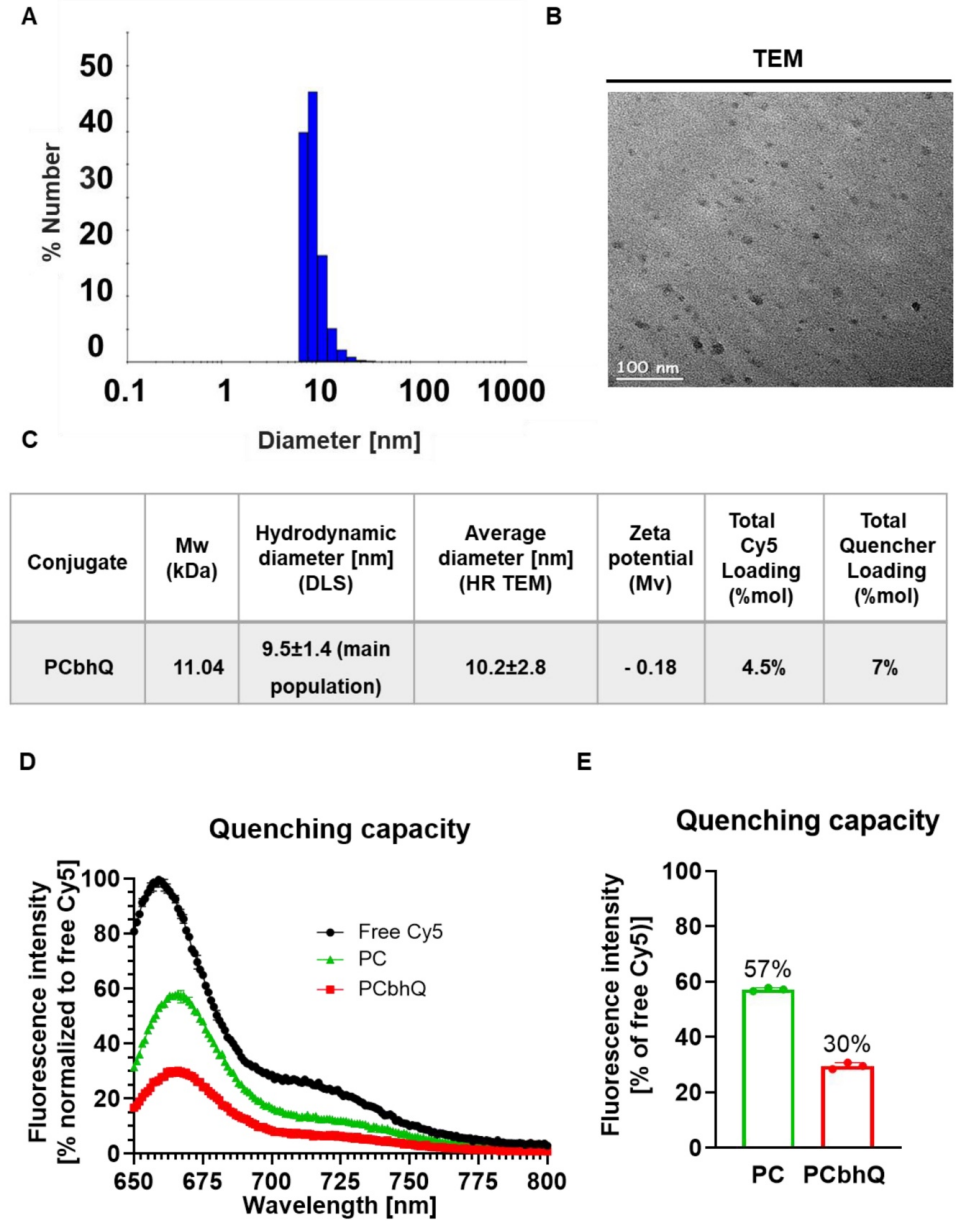
**Physico-chemical characterization and quenching capacity of PCbhQ Turn-ON probe. (A-C)** Physico-chemical characterization of the PCbhQ probe by (A) DLS and (B) High-resolution TEM. Summary of size, molecular charge and Cy5/BHQ loading (C). **(D)** Emission spectra of PCbhQ FRET-based probe in comparison with free Cy5 and SQ PC probe at 10 µM Cy5 equivalent concentration. **(E)** The fluorescence signal attenuation by different Turn-ON probes in comparison with free Cy5 (indicated as 100%). Data represent mean (n = 3 technical repeats).

The obtained probe was characterized by different modalities to determine its size and molecular charge. The mean hydrodynamic diameter of the main population (representing 99%) measured by Dynamic Light Scattering (DLS) was found to be 9.5 ± 1.4 nm (Figure 2A). This size was confirmed by High Resolution Transmission Electron Microscopy (HR TEM) measurements that showed an average diameter of 10.2 ± 2.8 (Figure 2B). The UV-Vis spectra of PCbhQ, PC, free Cy5 and free BHQ were collected (Figure S1D).

#### Quenching capacity evaluation of PCbhQ Turn-ON probe

Quenching capacity of the PCbhQ Turn-ON probe was evaluated in comparison with (i) PGA-Cy5 (PC) conjugate, in which the fluorescence signal attenuation is obtained due to self-quenching (SQ) of Cy5 molecules, and with (ii) free, unconjugated Cy5 (at equivalent concentration of 10 μM Cy5) which was defined as 100% signal. As expected, both SQ and FRET-based systems exhibited fluorescence signal attenuation in comparison with free Cy5. However, PCbhQ FRET-based system had the most pronounced effect of *circa* 70% signal reduction compared to 43% attenuation obtained by SQ PC conjugate (Figure 2D-E).

#### Turn-ON capacity of PCbhQ following enzymatic degradation with cathepsin

**Figure 3:**
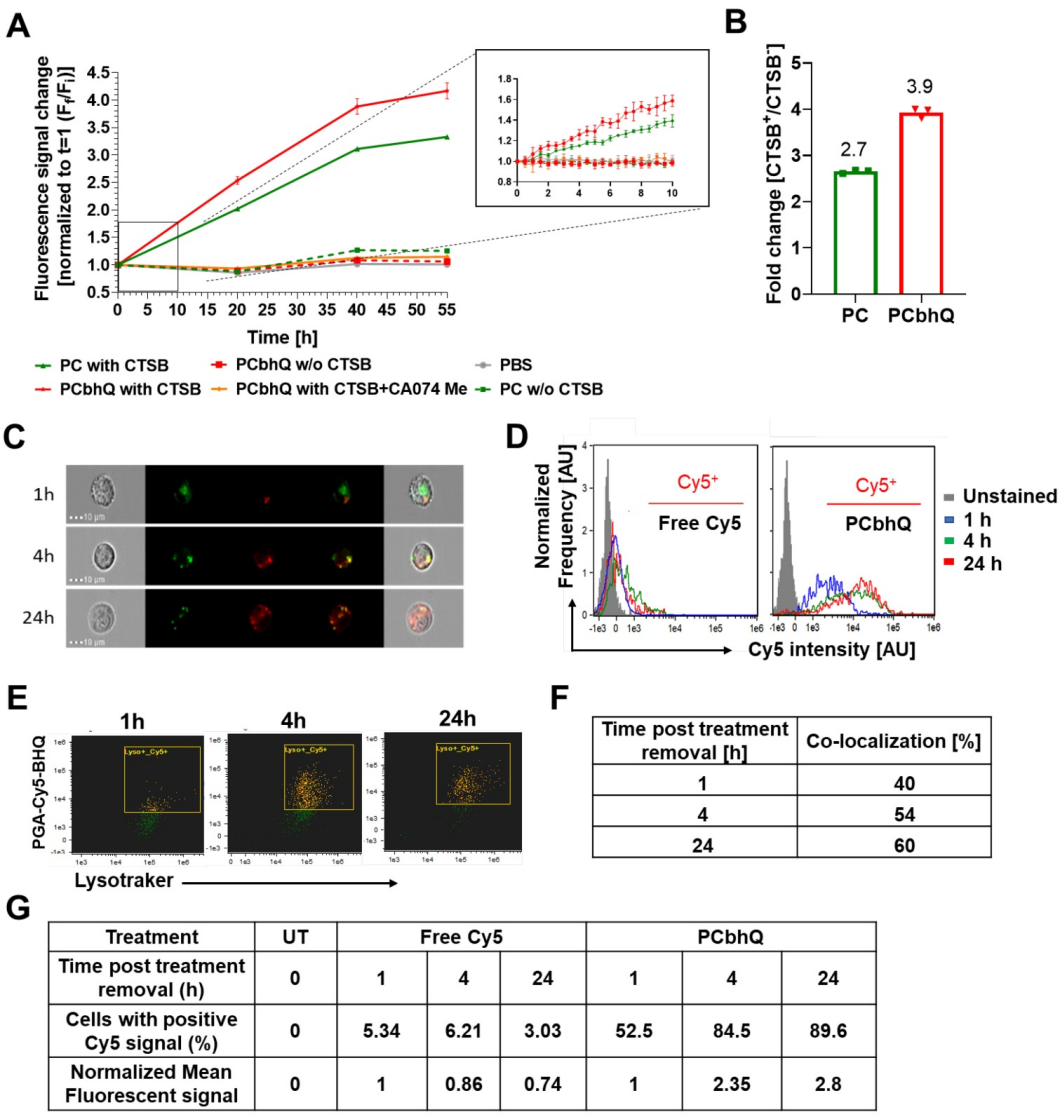
**Evaluation of enzymatic degradation *in vitro* and in living cells. (A)** Fluorescence signal elevation following PCbhQ (red) incubation with CTSB compared to that of SQ PC probe (green). PBS (grey), no enzyme (pH 5.5, dashed lines), and CTSB with CTSB inhibitor, CA-074 Me (orange) were used as a control. **(B)** The fluorescence signal fold change obtained following 55 h incubation of PC and PCbhQ in the presence or in the absence of CTSB at an acidic condition. Data represent mean ± SD (n = 3 technical repeats). **(C)** Cellular internalization and trafficking of PCbhQ (red) in living D4M.3A murine melanoma cells (lysosomes labeled in green), following 1 h, 4 h and 24 h incubation with PCbhQ Turn-ON probe. **(D)** Histograms representing fluorescence signal Turn-ON of PCbhQ following degradation of PGA backbone over time. D4M.3A melanoma cell were incubated for 1 h, 4 h and 24 h with PCbhQ Turn-ON probe or with the control, free Cy5. **(E and F)** Time-dependent co-localization (orange) of PCbhQ (red) with lysosomes (green) displayed by dot plot analysis of imaged living cells. **(G)** Mean fluorescence intensities and percentage of cells positive to Cy5 calculated from histograms.

In our previous work 46, we have validated the relevance of cathepsin B (CTSB) by demonstrating its over-activation in murine and human melanoma cells compared to that in normal skin. Here, to investigate the enzymatically-mediated Turn-ON abilities of our novel system, PCbhQ conjugate was challenged with CTSB. Following 55 h incubation, PCbhQ had a higher Turn-ON fluorescence effect than the SQ PC of 3.9 *versus* 2.7, respectively (Figure 3A-B). As a control, PCbhQ was incubated in acidic or neutral conditions in the absence of CTSB. A negligible change in fluorescence signal indicates its stability against degradation in these conditions (Figure 3A-B). Fluorescence spectra of PCbhQ and PC was collected during first 15 hour of incubation. The spectra are presented at the starting point of the incubation and following 15 hours (Figure S1E-I).

#### Concentration-dependent inhibition effect of CTSB inhibitor

Dose dependent activity of CTSB inhibitor, CA-074 Me, was evaluated by pre-incubation of PCbhQ probe with various concentrations of CA-074 Me (from ~0 μM to 250 μM) in the presence or absence of CTSB enzyme (0.2 U/mL). The degradation of PGA by CTSB was indeed inhibited in a dose-dependent manner. As the inhibitor concentration increased above 7 μM, the fluorescence signal emitted from the probe was decreased by 50% in comparison with its lowest concentration (Figure S2A-B). This suggests the dose-dependent competitiveness of the CA-074 Me inhibitor with CTSB for the affinity of the PGA-based probe.

#### Cellular uptake, co-localization with lysosomes and Turn-ON abilities of PCbhQ

To validate the intracellular trafficking and Turn-ON ability of our novel PGA-based probe, live-cell imaging of D4M.3A murine melanoma cells was performed in its presence. In order to track the intracellular trafficking rather than probe accumulation, PCbhQ was incubated with cells for 0.5 h then exchanged with fresh medium. The threshold for positive Cy5 signal was determined by the unstained cells. Our PCbhQ probe was successfully internalized into the cells, retained there, and showed time-dependent fluorescence signal increase up to 2.8-fold from t_0_ to 24 h post incubation (Figure 3C-D) and with larger Cy5-positive population in comparison with cells that were incubated with free Cy5 (Figure 3G). This is in correlation with its gradual co-localization (Figure 3E, orange pixels) increase from 40% to 60% with lysosomes (Figure 3F). The yellow pixels in Figure 3E indicate cells with green (stained by LysoTracker® Green) and red (PCbhQ) staining that are not in co-localization, and green indicates non-gated cells.

#### *In vivo* characterizations of PCbhQ

**Figure 4:**
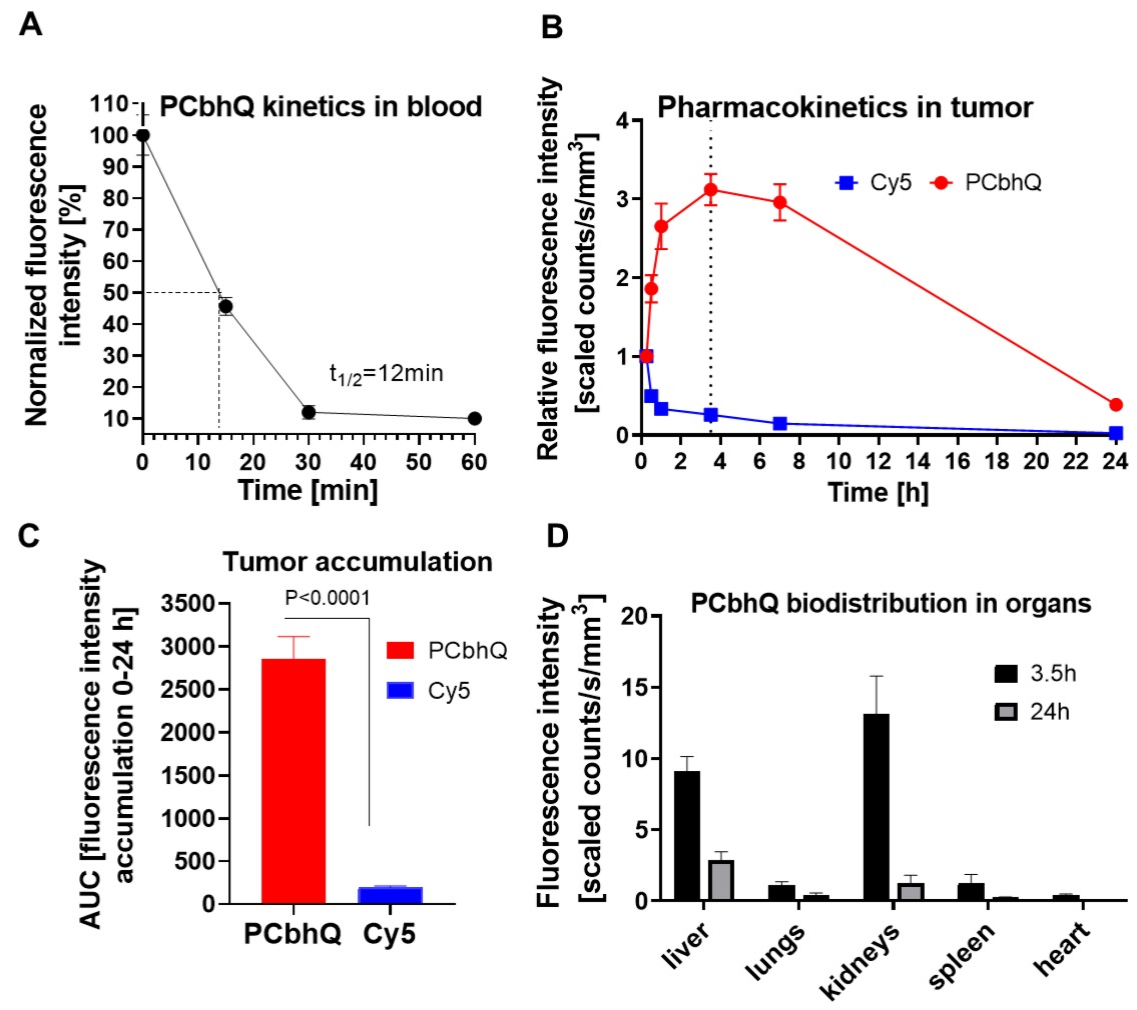
***In vivo* characterizations of PCbhQ. (A)** Pharmacokinetic evaluation of PCbhQ Turn-ON probe in blood up to 60 min post *i.v.* administration. T_1/2_ =12 min. (N = 3, each point represents mean ± SEM). **(B)** Pharmacokinetic evaluation of PCbhQ Turn-ON probe in tumor up to 24 h post *i.v.* administration. Maximum accumulation of the probe was observed after 3.5 h. Free Cy5 accumulation in the tumor was measured as a control (N = 4-13, each point represents mean ± SEM). **(C)** Total tumor accumulation (AUC) of PCbhQ and free Cy5 during 24 h evaluation (each bar represents mean ± SEM). **(D)** Biodistribution in different organs of PCbhQ after 3.5 h and 24 h post *i.v.* administration. (N = 5, each bar represents mean ± SD).

To evaluate the pharmacokinetics of the probe in blood, PCbhQ (40 µM Cy5-eq concentration in 200 µL PBS) was injected *i.v.* into D4M.3A melanoma-bearing mice. At predetermined intervals blood samples were collected and the fluorescence signal in the plasma was evaluated. T_1/2_ of the probe was obtained to be 12 min. Negligible fluorescence signal was detected 1 h post probe administration (Figure 4A). This corresponds with the results obtained from the 24 h, non-invasive fluorescence signal evaluation in the tumor following PCbhQ and free Cy5 *i.v.* injection into D4M.3A melanoma-bearing mice. Signal elevation with time was observed in mice following PCbhQ administration compared to mice that received free Cy5. Maximum signal accumulation was presented 3.5 h after PCbhQ administration. Importantly, the prolonged retention of the probe in the tumor, which could be attributed both to its' accumulation and Turn-ON abilities was presented up to 7 h (Figure 4B). Total tumor accumulation of the probe obtained during 24 h evaluation was significantly higher than free Cy5 accumulation (Figure 4C).

The evaluation of the biodistribution of PCbhQ nanoprobe 3.5 h post administration showed its' accumulation mostly in kidneys and partially in liver with a significant drop of it in both organs 24 h after the administration. No significant accumulation was observed in any other of examined organs (Figure 4D).

#### Primary tumor resection with PCbhQ optical guidance

**Figure 5:**
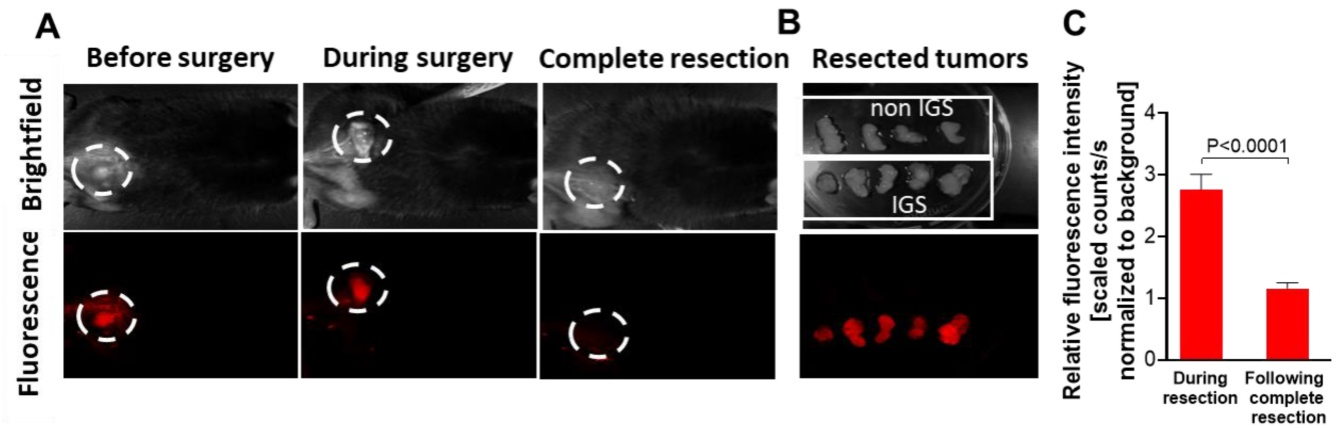
** Primary tumor resection under PCbhQ fluorescence guidance. (A)** Representative images from the surgical field during surgery performed under PCbhQ guidance. **(B)** Tumors resected with or without imaging guiding. **(C)** TBRs obtained during IGS and post complete tumor resection. (*P* < 0.0001, two-tailed Student's t-test). Data represent mean ± SEM (N = 20).

PCbhQ (40 µM in 200 µL PBS) was injected *i.v.* into D4M.3A melanoma-bearing mice that were randomized for IGS, about 3.5 h before the surgery. This time point was selected in accordance with fluorescence signal kinetics evaluated following PCbhQ *i.v.* administration (Figure 4B). The fluorescence guidance was performed using CRI Maestro^TM^ imaging system. Figure 5A upper panel shows the brightfield images of tumor and tumor bed. The lower displays the corresponding fluorescence images. Tumor tissue was excised according to the fluorescence signal. The resection was defined as completed when the signal in the surgery site was as low as the signal measured from the adjacent areas. Tumor to Background (TBR) during tumor excision was found to be almost 3 times higher than the TBR following complete excision (Figure 5C). In the control group, tumors were resected under white light, without the PCbhQ guiding probe. All resected tumors were imaged after removal (Figure 5B). Both groups were monitored for tumor recurrence and wellbeing. We found that 10 days post tumor resection, 33% of mice from the control group experienced primary tumor recurrence compared to only 23% of mice that underwent IGS. This may suggest that when the operation is optically-assisted, a more precise resection of the tumor is obtained.

#### Evaluation of safety profile of PCbhQ

To test the biotoxicity of PCbhQ, we followed the weight change of mice and performed blood count and blood chemistry following its administration to mice. In addition, we assessed the *in vitro* effect of the probe on cell viability of NIH-3T3 normal fibroblasts. No toxic effects *in vivo* or *in vitro* were identified (Figure S3A-C).

### Post-surgical, adjuvant treatment and follow-up as a second therapeutic step

To mimic the clinical scenario where cancer cells evade the primary tumor and circulate the body, we injected intracardially melanoma cells and treated the mice with our novel targeted, polymeric system (PGA-SLM-mDBF), previously described by our lab 43 combined with immunotherapies (ICPi consisting of αPD-1 and αCTLA-4 antibodies) before metastases were detected by imaging evaluation. This prophylactic therapy was evaluated as a second step treatment following surgery, to prevent post-surgical tumor recurrence and delay or prevent metastases formation. In addition, the safety profile of the combined treatment was evaluated. To evaluate the long-lasting effect of the different therapies, we continued monitoring the treatment efficacy using our Turn-ON probe, PCbhQ, following treatment termination.

#### PGA-SLM-mDBF conjugate targeting MAPK pathway in BRAF V600E-mutated melanoma

Here, we evaluated the potential of PGA-SLM-mDBF as a preventive, anti-metastatic, post-operative therapy. To that end, PGA-SLM-mDBF conjugate was designed and synthesized as previously described 43. Prior to conjugation, DBF was functionalized with a diol group to become modified DBF-diol (mDBF). The final conjugate structure and physico-chemical characterization summary are depicted (Figure S4A-B)**.**

#### PGA-SLM-mDBF conjugate alone or in combination with ICPi decrease D4M.3A melanoma tumor spheroids sprouting

**Figure 6:**
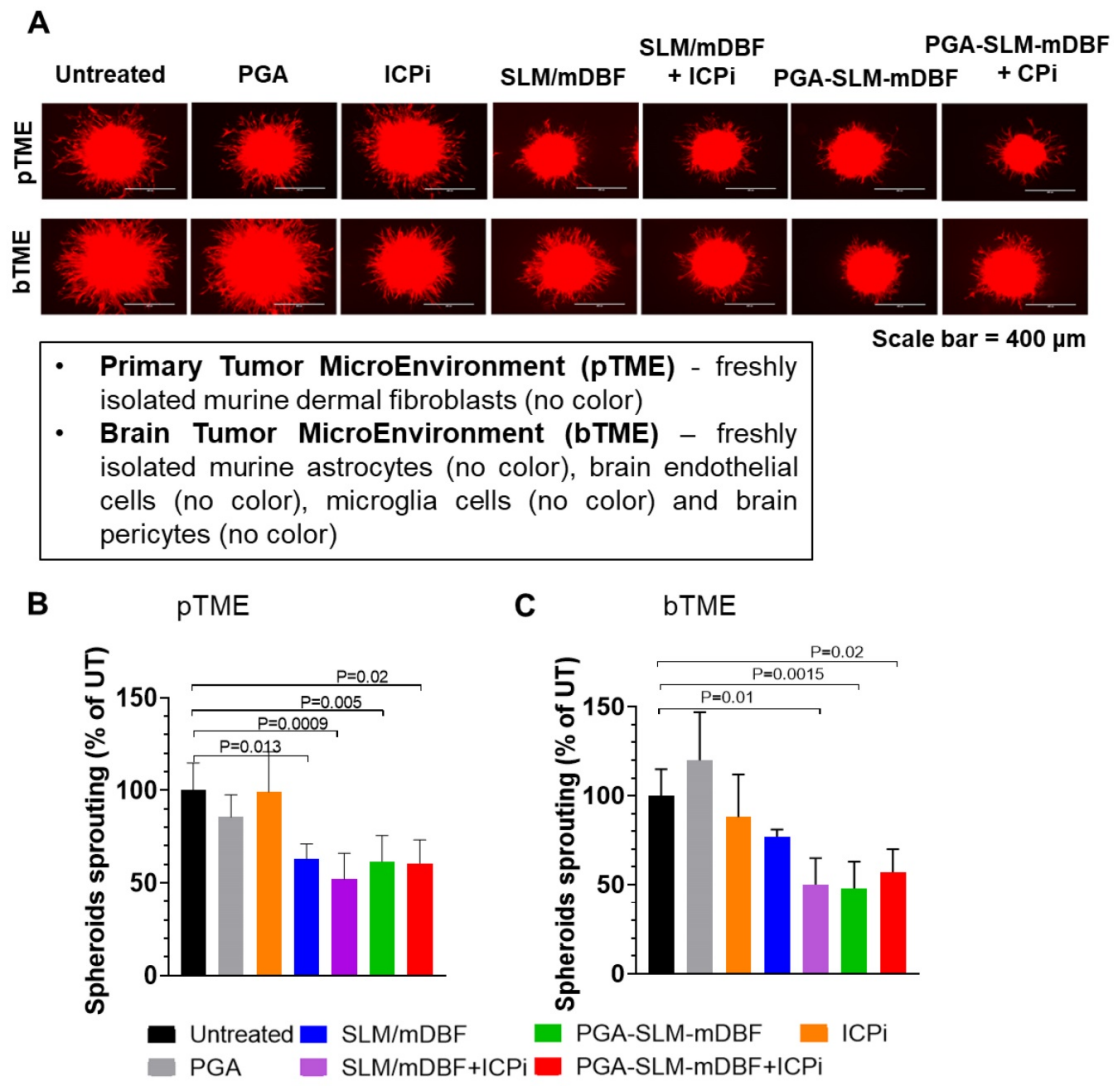
** PGA-SLM-mDBF inhibits D4M.3A melanoma 3D spheroids sprouting. (A)** Mimicking 3D primary tumor and brain microenvironment, (pTME and bTME, respectively) by tumor spheroids of D4M.3A-mCherry, murine melanoma (red) and freshly isolated cells (no color) as indicated in the insert. Scale bar = 400 μm. The tumor spheroids were either untreated or treated with PGA nanocarrier, SLM/mDBF (free drugs combination), PGA-SLM-mDBF conjugate or conjugated and non-conjugated drugs in combination with ICPi. Melanoma cell sprouting was evaluated after 24 h. **(B-C)** Quantification of D4M.3A melanoma mCherry signal in tumor spheroids of pTME (B) bTME (C) P values are indicated in the graphs, N = 3-8/each treatment group, one-way ANOVA with Turkey's multiple comparisons test.

To further potentiate the therapeutic efficacy, a combination of two ICPi, αPD-1 and αCTLA-4 antibodies, were added to the treatment schedule. First, we evaluated the effect of our combined PGA-SLM-mDBF conjugate alone or with the combination of ICPi on our unique 3D multicellular immune-activated melanoma model. Both PGA-SLM-mDBF or its combination with ICPi significantly inhibited melanoma cell sprouting compared to untreated control (PGA-SLM-mDBF: *P =* 0.005 in case of primary tumor microenvironment (pTME), *P =* 0.015 in case of brain metastasis tumor microenvironment (bTME) and PGA-SLM-mDBF+ICPi: *P =* 0.02 tested either in pTME or in bTME) (Figure 6). This effect was similar to the effect obtained following treatment with free drugs combination with ICPi. However, in the presence of bTME, the effect of the unconjugated free drugs alone was less pronounced.

#### Evaluation of melanoma tumor growth inhibition by PGA-SLM-mDBF conjugate in combination with ICPi

**Figure 7:**
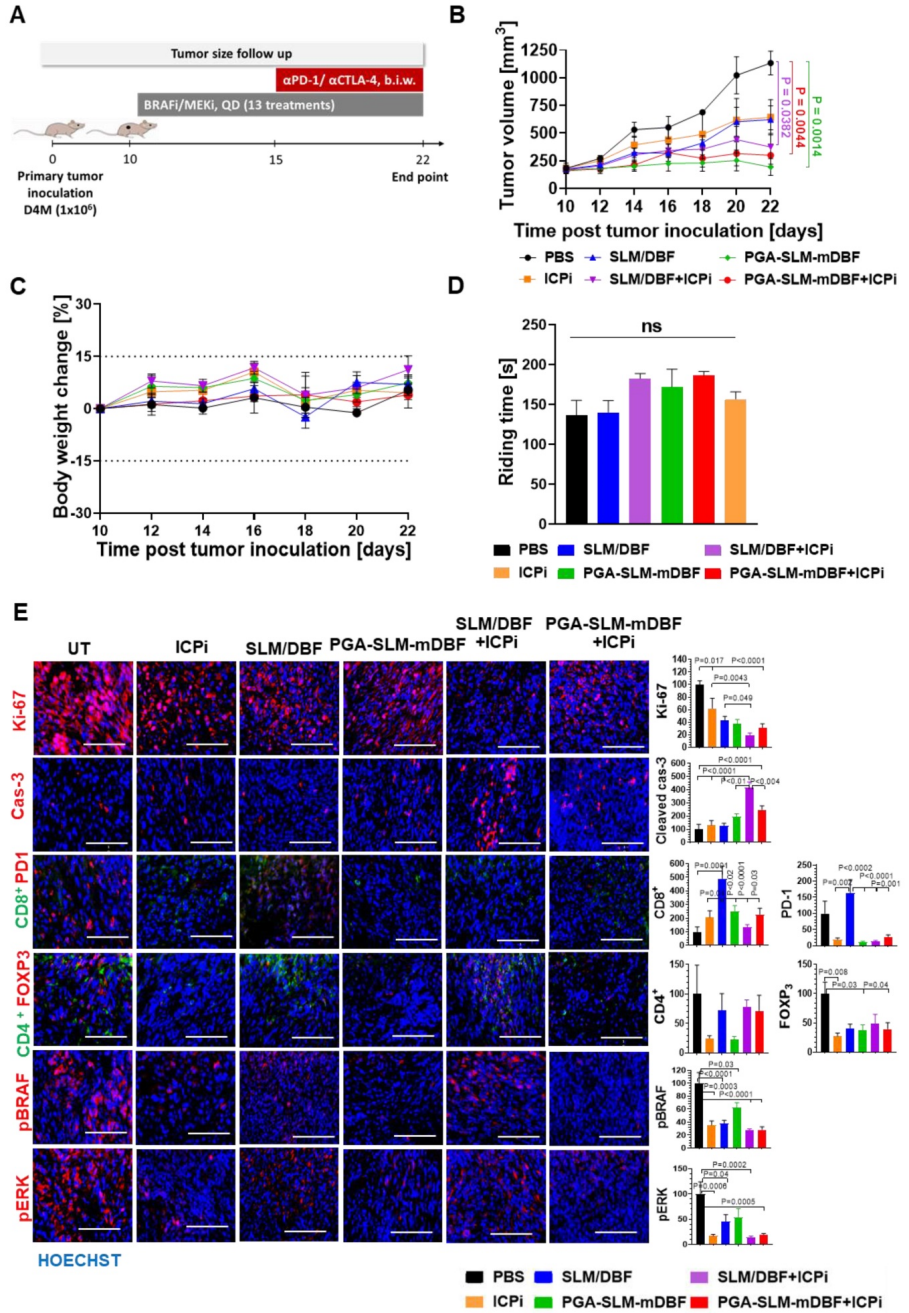
** Antitumor activity and safety profile of PGA-SLM-mDBF in D4M.3A melanoma-bearing mice. (A)** Timeline (days) of tumor inoculation, treatments, and follow-up. **(B)** Tumor volume growth curve. **(C)** Body weight change. **(D)** Mean mice riding time ± SEM during rotarod performance test. N = 5 mice per group. (*P* < 0.05). **(E)** Histological analysis of D4M.3A tumors. Representative microscope images (left) and quantification (right) of D4M.3A tumor section stained for proliferation, apoptosis and MAPK pathway markers as well as for tumor-infiltrating immune cells. Y-axes values represent (% of total area normalized to untreated cells). N = 3 samples per group. Scale bar is 100 μm for all images. Statistical significance for (D) and (E) was determined using one-way ANOVA test with multiple comparisons adjustments (*P* < 0.05).

D4M.3A melanoma-bearing C57LB/6 mice were treated for 13 days, with DBF and SLM, free or conjugated to PGA (at DBF-equivalent concentration of 10 mg/kg) alone or in combination with ICPi (αPD1 and αCTLA-4 antibodies, 5 mg/kg each). One group was treated with ICPi alone, while the control group was injected with PBS. As expected, PGA-SLM-mDBF alone or in combination with ICPi inhibited the tumor growth more effectively (*P =* 0.0014 or *P =* 0.0044, respectively, compared to the control group), maintaining stable tumors volume during the treatment course. Our conjugate alone was found to be more effective than the free drugs alone or in combination with ICPi, or ICPi only. However, it can be discerned that although initially ICPi addition did not show added value compared to any of the treatments, it started to be pronounced in mice treated with ICPi in combination with free drugs cocktail on day 20, which is around 6 days after ICPi treatment initiation (*P =* 0.0382 compared to PBS group) (Figure 7A-B).

#### Evaluation of the safety profile of free and PGA-conjugated BRAF/MEK inhibitors

To address the possible toxic effects of the treatments, mice from all treatment groups were routinely weighed and their blood chemistry and hematology were evaluated 24 h after the last treatment. All mice presented a stable weight change curve (Figure 7C). Moreover, blood analysis showed that PGA-SLM-mDBF conjugate did not cause any significant changes in liver and kidney functions, blood chemistry values or blood count. This is in contrast to mice that were treated with free drugs that exhibited a certain increase in the majority of these parameters (Figure S5). These findings stand in line with polymer therapeutics theory hypothesizing that the conjugation of the drugs to polymeric nanocarriers results in a more precise delivery of the drugs to the tumor that consequentially lead to decreased toxicity of the drugs.

Drugs' intoxication may affect animals' behavior, particularly coordination and learning abilities 51. Thus, to assess the motor coordination of mice from different treatment groups, rotarod performance test was performed. Evaluation of mice ability to walk forward over time without falling off showed that there was no significant difference between the groups, suggesting that none of the treatments caused neurotoxicity nor motor coordination and imbalance impairment at the administered doses (Figure 7D).

Immunohistochemical evaluation of frozen tissue sections was performed to trace the molecular mechanism of the tumor growth inhibition *in vivo*. All treatments led to significant decrease in proliferation (Ki-67), however, the combination of free or PGA-conjugated drugs with ICPi exhibited the most pronounced effect. In a similar manner, these treatments showed the highest apoptotic effect. MAPK pathway evaluation revealed a marked decrease in the expression level of the phosphorylated form of key pathway-related enzymes, BRAF and ERK1/2 following treatments in comparison to the untreated group, supporting their involvement in tumor growth inhibition. To evaluate the impact of the treatments on immune infiltrates, tumor tissue sections were stained for T_eff_ and T_reg_ (CD8^+^ and CD4^+^/FOXP3) subsets. All treatments caused some extent of increase in CD8^+^-T cell infiltration into the tumor site, while the most prominent effect was obtained in the free drugs-treated group. Moreover, a decrease in T_reg_ cells population (CD4^+^/FOXP3) and PD-1 was detected in all treated groups besides the higher PD-1 level indicated in free drugs-treated group (Figure 7E).

#### Evaluation of the ability of PGA-SLM-mDBF alone or in combination with ICPi to prevent metastasis development

**Figure 8:**
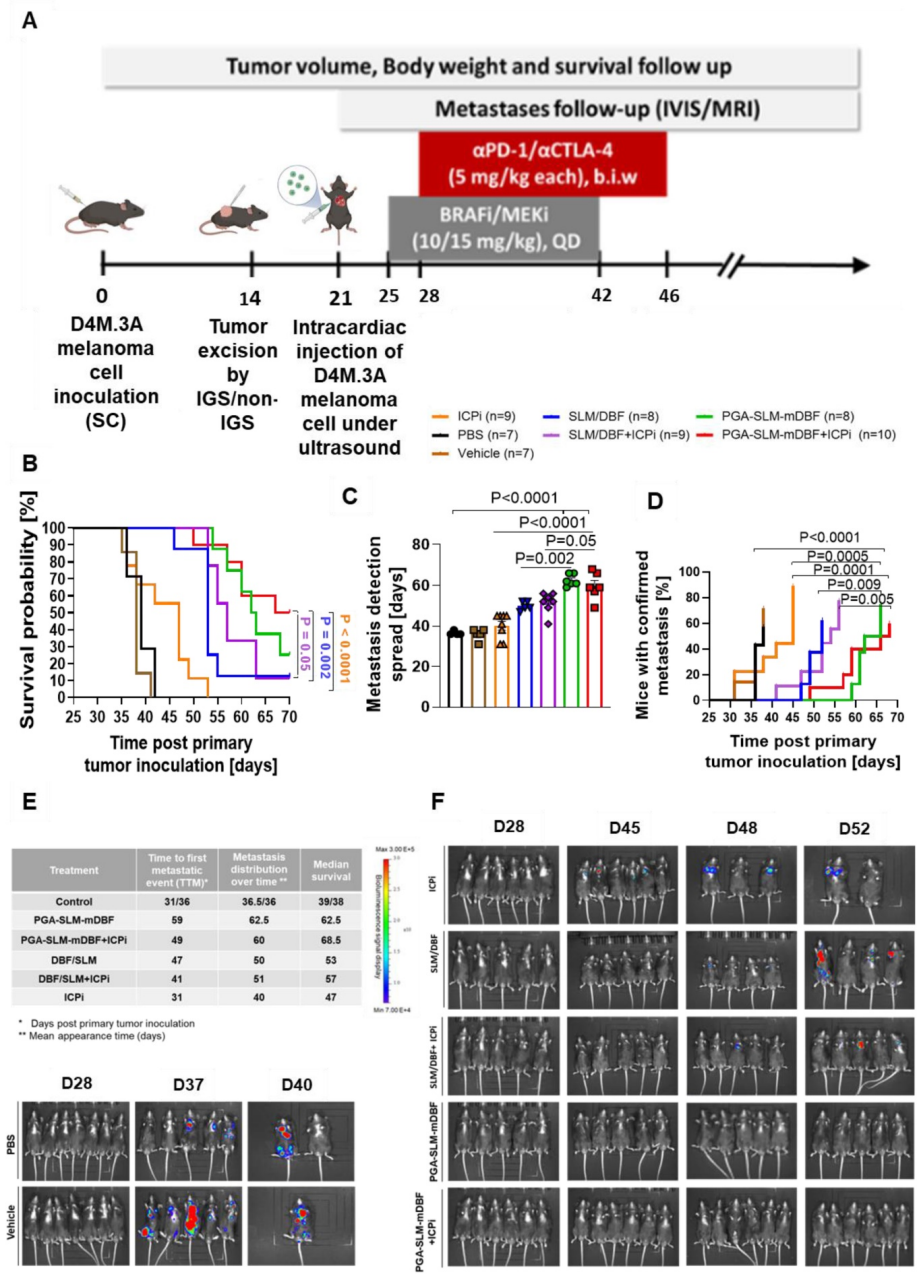
** Preventive, anti-metastatic activity of PGA-SLM-mDBF conjugate alone or in combination with ICPi targeting PD-1 and CTLA-4 in D4M.3A murine metastatic melanoma model. (A)** Timeline (days) of the experiment. **(B)** Kaplan-Meier survival curves of the different treatment groups. **(C-D)** Metastases development inhibition, presented as the kinetics of development and distribution over time inside the groups. **(E)**. Tabulated summary of the survival and metastases development data. **(F)** Representative images of mice monitored over time with IVIS® Lumina III *in vivo* Imaging System. Data represent mean ± SEM. Statistical significance was determined using one-way ANOVA test with multiple comparisons adjustments (C) and using survival comparisons (B) and (D) (*P* < 0.05). Number of mice in each group is indicated in the relevant panel.

We performed *in vivo* evaluation of the ability of PGA-SLM-mDBF, alone or in combination with ICPi, to prevent post-surgical tumor recurrence and metastasis development. In order to induce metastases, ultrasonography-guided intracardiac injection of D4M.3A luciferase-labeled cells was performed into the arterial circulation of C57BL/6 mice. Treatment groups and experimental timeline are indicated in the scheme (Figure 8A). Control mice were treated with either PBS or with the vehicle which was used for the solubilization of the free drugs. PGA-SLM-mDBF conjugate alone or in combination with ICPi was the most efficient in prolonging the mice's overall survival (OS). The conjugate alone extended the median survival of the mice to 62.5 days compared to PBS-treated mice with a median survival of 39 days (*P =* 0.0002). The combination of the conjugate with ICPi resulted in even more pronounced effect prolonging mice median survival to 68.5 days (*P <* 0.0001). Importantly, this combination treatment was highly effective compared to mice treated either with free drugs, ICPi alone, or with free drugs + ICPi combination that presented median survival of 53, 47 and 57, respectively (*P =* 0.002, *P =* 0.05 and *P <* 0.0001, respectively) (Figure 8B). Next, we characterized the distribution of metastases in each group over time.

Again, mice treated with PGA-SLM-mDBF or with its combination with ICPi presented significantly delayed average metastases appearance time (62.5- or 60 days, respectively) compared to other treatment groups (Figure 8C). The average appearance time for mice treated with free BRAFi/MEKi alone or in combination with ICPi was 50 or 51 days, respectively, and for the ICPi alone and the control treated group was 40- and 36-days post injection, respectively. Furthermore, treatments with PGA-SLM-mDBF alone or in combination with ICPi were superior to all other treatments in preventing melanoma metastases development, postponing the time of the first confirmed metastatic event (TTM-time to metastasis) from day 31/36 post primary tumor inoculation observed in control groups to day 49/59 for conjugate plus ICPi or conjugate alone treated groups, respectively (Figure 8D). Also, metastases development kinetics inside these groups was slower compared to all other groups, as indicated by the time at which 50% of the mice in the group (from those who eventually developed) presented with metastases.

Data summarizing all groups' survival and metastasis development course together with representative images from IVIS® Lumina III *in vivo* Imaging System are shown (Figure 8E-F).

#### Metastasis and tumor recurrence follow-up with PCbhQ Turn-ON probe

To evaluate the long-lasting effect of the different therapies, we continued monitoring the treatment efficacy following treatment termination. In addition to the bioluminescence imaging, mice general health and neurological conditions were monitored. The first evidence for brain metastasis was recorded around day 60 post primary tumor inoculation (21 days post treatment termination). Representative images of brain tumors confirmed by MRI imaging are presented (Figure S6).

**Figure 9:**
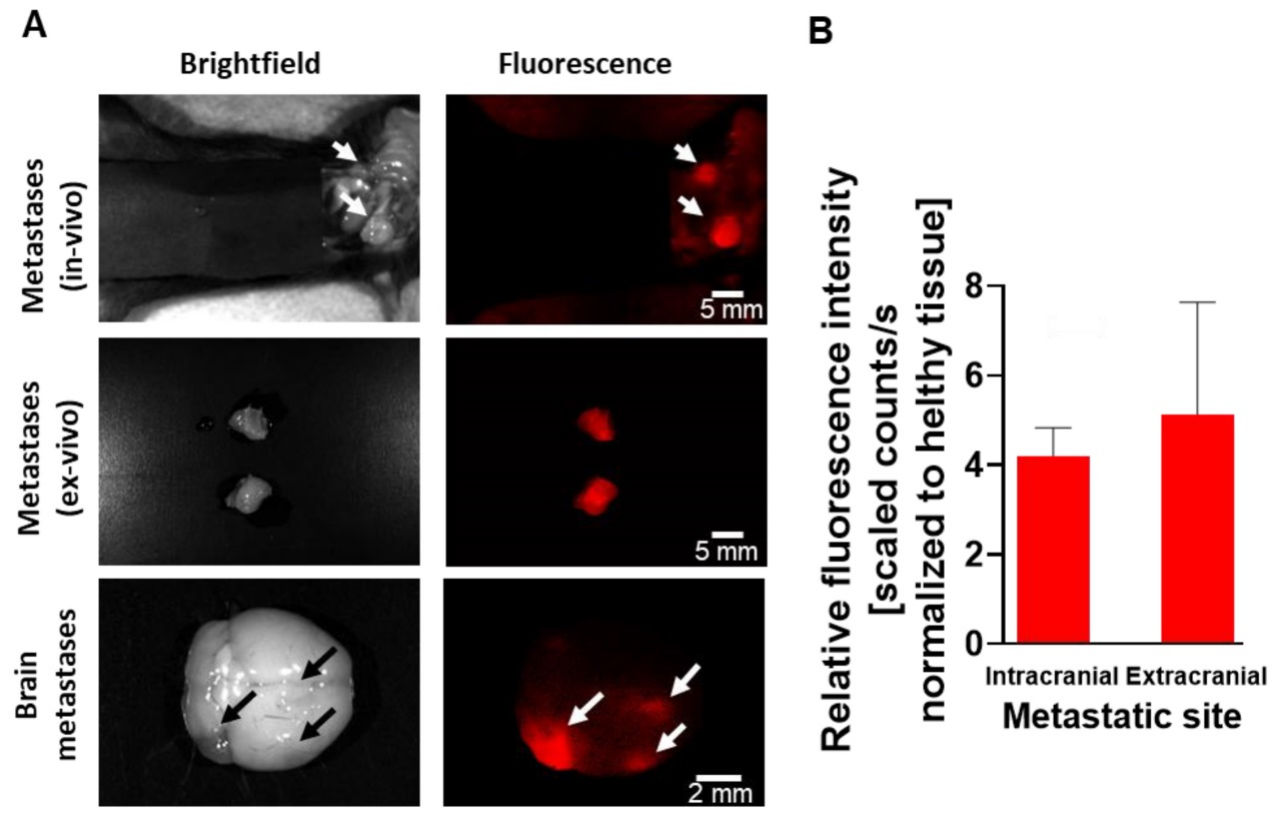
** The ability of the PCbhQ Turn-ON probe to detect small metastatic spread. (A)** Metastatic lesions in different organs (peritoneal cavity and brain). Bright field *versus* fluorescence visualization of the metastasis with PCbhQ (40 µM in 200 µL PBS), 3.5 h post *i.v.* administration, using CRI Maestro^TM^ imaging system. Scale bars of 2-5 mm are indicated inside the figure.** (B)** TBR obtained during the metastases imaging process. Data represent mean ± S.D. (N _Extracranial_ = 10, N _Intracranial_ = 5).

To evaluate the ability of our Turn-ON imaging probe to detect small metastatic lesions, selected mice, that reached the exclusion criteria (*i.e.* reached the endpoint), were fluorescently imaged by CRI Maestro^TM^, 3.5 h following PCbhQ *i.v.* administration. The probe succeeded to detect the metastatic spread located both in the abdominal cavity and in the brain, with spatial resolution of less than 1000 µm (Figure 9A). Importantly, one of the presented lesions could be detected only with fluorescence assistance and not during a white light examination. An average TBR of 4.2 for brain metastases detection and of 5.13 for extracranial lesions was determined (Figure 9B).

## Discussion

In this study, we present the design and preclinical implementation of a two-step treatment, based on the PGA nanocarrier synthesized in our laboratory. PGA is a multivalent, biodegradable and biocompatible, well-studied, safe and efficient polymeric platform 52-54.

The first step, IGS of the primary tumor was performed with a novel 'smart' polymeric near infrared (NIR) FRET-based Turn-ON probe, PCbhQ. Fluorescence imaging, in the far-red range or above allow higher depth of penetration 46, 55 thus enabling better intraoperative assessment of tumor boundaries in real-time. Another important merit for surgical procedure is sufficient imaging duration by the guiding probe. Low molecular weight probes for cancer surgery navigation include targeted, cell-penetrating peptides and activatable substrates that make use of enzymes for fluorescence activation 56-58. However, most of them have a short plasma half-life, sometimes insufficient accumulation inside tumor cells and relatively rapid bleaching 59-62. These limit their application for prolonged procedures as complicated surgeries. To overcome these obstacles, 'smart' nanoprobes with activation mode of action have been reported for *in vivo* imaging. Among them are polymeric nanocarrier-bound, biomarker-sensitive conjugates 63, quantum dots 64 and fluorescently-tagged targeted antibodies 65.

We have previously explored and showed the advantages of conjugating drugs and imaging agents to polymeric platforms 43, 44, 46. In our previous study, two PGA-based biodegradable imaging probes were evaluated and compared with HPMA copolymer-bound probe with a different mode of activation 46. Based on our findings from this study, striving to enhance the quenching efficiency and Turn-ON capacity for *in vivo* implementation, Cy5 was paired with a different quencher molecule, BHQ dark quencher, on one PGA polymeric platform. Changing the quencher molecule and increasing the loading of the dye and quencher was conveniently allowed by binding to the glutamic acid monomers on the PGA carrier and resulted in smaller hydrodynamic diameter of the probe (9.5 nm) compared to 150 nm in our previously-reported Turn-ON probes 46. Importantly, this size (< 10 nm) is reported as ideal for imaging purposes 66, 67. This decreased size might derive from increased π-π stacking interactions and different spatial molecular orientation. We believe that the smaller size of our new PCbhQ probe resulted in a shorter time gap of 3.5 h between *i.v.* injection and imaging, in contrast to our former PGA based Turn-ON probe, named PCQ, that had a time gap of 24 h post *i.v.* injection. It is worth mentioning that this achievement is best shown in the *in vivo* setting, as several proteases that are known to degrade PGA to some extent, such as Cathepsin B, S, L and others 46, 68 are present in the tumor microenvironment. This is in contrast to the *in vitro* setting, when PCbhQ was challenged with CTSB only to show its enzymatic degradation abilities. We assume that this may be the reason for the longer kinetics of degradation *in vitro* versus *in vivo,* reaching a maximum signal 55 h post-incubation with CTSB, *versus* 3.5 h post i.v. injection. Shorter injection-to-procedure time gap is desired for minimizing hospitalization and hence, decreasing insurance costs and increasing patient compliance. Self-quenched probes such as ProSense® 680 are typically made from high-molecular-weight polymeric carriers that require a long post-injection lag time 69. Other recently reported FRET-based probes present longer time to achieve a sufficient TBR, ranging from 24 to more than 100 h 70. In comparison with our formerly reported SQ Turn-ON probe (P-GFLG-Cy5), PCbhQ presented a very similar quenching capacity (“OFF” state) of 70% *versus* 73% reduction in the fluorescence signal compared to the signal obtained from the free Cy5. However, it presented a higher Turn-ON capacity (“ON” state) *in vitro* (3.9 *versus* 2.7-fold-change from “OFF” state), which indicates 44% increase in fluorescence signal post probe activation. Importantly, *In vivo* evaluation of our probe, bearing 7 mol % of BHQ molecules, resulted in an average TBR of 2.8 that allowed to clearly detect the tumor boundaries 3.5 h following the probe injection to the mice. This shows a similar increase of 47% for our novel PCbHQ probe in comparison with our former SQ-based probe for *in vivo* imaging of the tumor *versus* the healthy background (2.8 *versus* 1.9 TBR, respectively) 46. The TBR obtained from the tumor bed after tumor resection was decreased to *circa* 1, that was indicated by us as no evidence for residual disease left behind. This 2.8-fold change between cancerous and background healthy tissue stands in line with other fluorescent Turn-ON probes, that were clinically evaluated, such as ratiometric protease-activatable AVB-620 15 and higher than that of LUM015, a cathepsin-activatable polymeric probe that showed a 1.8 tumor-to-skin ratio on murine colorectal cancer model 71. Finally, we showed that IGS performed with the guidance of PCbhQ resulted in a lower recurrence rate (23%) of the primary D4M.3A tumors in comparison with the control group (33%) of mice that underwent surgery under white light, suggesting the ability of the probe to promote a better patient's outcome. Similarly, Zhao *et al.* showed no recurrence for 72% of mice that underwent IGS by a tumor-acidosis guidance probe 72. Notably, recent clinical validation of IGS by diverse imaging probes is ongoing and investigates whether the incidence of positive tumor margins is decreased 11-15. Among these imaging probes are fluorescent tumor-targeted probes such as Tumor Paint, penetrating tumor cells by means of chlorotoxin protein 11, folate receptor-targeted fluorophore EC17 12, vascular endothelial growth factor (VEGF)‐targeted fluorescent tracer 13, and activatable Turn-ON probes such as the cathepsins and the MMPs activatable probes, LUM015 14 and AVP‐620 15, respectively.

The biodistribution of PCbhQ shows increased fluorescence signal in the liver and kidneys. Hence, a potential limitation of our Turn-ON PCbhQ is that it may not be suitable for imaging of tumors that are adjacent to liver or kidneys as these are the main excretion organs of the body, and thus might result in a fluorescence signal that is not necessarily related to the presence of a cancerous tissue. Accordingly, we imaged the surgery at the region of interest, away from these organs, similarly to a standard image-guided surgery settings in clinical trials.

However, despite advanced surgical technologies, the tumor cells may spread to secondary organs even before or during the surgery, leading to aggressive melanoma within years from excision. It is demonstrated by the rising number of patients presenting metastatic melanoma in accordance with the disease stage, that reaches up to 75% at the time of death 4, 73-75. Thus, to potentiate the chance for complete disease eradication, we proposed here a precise tumor resection followed by effective post-surgery, adjuvant therapies for the prevention of tumor recurrence and metastases. Targeted therapies and recently erupted immunotherapies 76, possessing distinctly different mechanisms of action, both found to be effective in patients with advanced disease 77, 78. Combining these two therapeutic approaches led to higher response rates and prolonged durability 79. In our recent study 43, we introduced the polymeric therapeutic platform, PGA-SLM-mDBF, simultaneously delivering SLM (MEKi) and modified DBF (mDBF) (BRAFi). This platform was able to improve substantially the antitumor activity and OS of primary melanoma-bearing mice while reducing the dose by up to 3-fold compared to the free drugs in previous reports 80, 81. In addition, multiple studies have demonstrated a favorable TME emergence, including increased melanoma antigen expression, tumor-infiltrating CD8^+^ T cells (TILs) and other immunogenic cytokines signaling following the MAPK pathway inhibition with BRAFi/MEKi 79, 82, 83. Recognizing the potential for improved antitumor activity, we combined our targeted polymer-based therapy with ICPi (αPD-1/αCTLA-4) for metastases prevention.

In the current study we did not see pronounced beneficial effect of ICPi addition to the PGA-SLM-mDBF on the primary tumor growth during the first 22 days post tumor inoculation. We attribute this to the prolonged inhibition of tumor growth by PGA-SLM-mDBF. This stands in line with the results presented previously at Pisarevsky et.al, that showed a prolonged effect of PGA-SLM-mDBF on the inhibition of melanoma growth for up to 35 days post primary tumor inoculation 43 which is 13 days post treatment termination. On the other hand, the trend among the groups treated with an unconjugated (free) BRAFi/MEKi, which showed tumor growth escape much earlier is much more pronounced, with clear superiority to the group treated with drugs combined with ICPi, 6 days post ICPi treatment initiation (20-day post tumor inoculation). Accordingly, the beneficial effect of the ICPi combined with our PGA-SLM-mDBF is shown in the setting of metastases inhibition. We demonstrated that a combination produced prolonged metastases-free survival (MFS) and OS in the intracardially-induced murine melanoma model, in comparison with free BRAFi/MEKi alone or in combination with ICPi or ICPi alone. As a preventive approach, we used here reduced doses of BRAFi/MEKi (10 mg/kg for BRAFi and 15 mg/kg for MEKi) 43, and of ICPi (5 mg/kg each), as opposed to other reports on mouse models administered with doses such as 30 mg/kg DBF (as part of a combination treatment) 80 and 10 mg/kg of ICPi 84. Moreover, the biodegradability of our PGA-based Turn-ON probe makes it safer for patients with co-morbidity of kidney diseases.

Aiming to mimic the clinical disease setting, we first injected orthotopically the murine D4M.3A melanoma cells, which bear the BRAF V600E mutation, into C57BL/6J mice. Tumor excision was done randomly with or without the guidance of our PCbhQ Turn-ON probe. Based on previous reports 85, we aimed to first activate the mice immune system with the primary tumor, and then, when the immune reaction has already initiated, to introduce peritoneal and brain metastases by intracardiac injection of luciferase-labeled D4M.3A cells. The treatment started 3 days post metastases induction, prior to their colonization, as confirmed by intravital bioluminescence imaging, to mimic the settings of metastatic prevention, rather than treating existing ones. Mice assigned to the combined targeted and immunotherapy group started to receive the ICPi supplement 3 days post BRAFi/MEKi treatment initiation, to allow the immune system induction first. Our system showed durable delay in metastases formation, even post treatment termination. A recent work of a combination treatment sequence of 1^st^ MEKi and 2^nd^ αPD-1 (10 mg/kg) inhibited the growth of murine primary CT26 colon tumor when given for 61-68 days, while follow-up period was 70 days 86. However, our polymeric based combined treatment of PGA-SLM-mDBF and ICPi was given for only 17 days (day 25-42) and 19 days (day 27-46), respectively, and showed prolonged survival until day 70, which is 24 days without treatment. Hence, our combined nano-sized treatment showed a better long-lasting inhibitory effect against metastases spread, while using shorter treatment time and lower dose.

In accordance with the clinically evident situation mentioned above, mice that survived longer after treatment termination developed brain metastases more frequently compared to short-term survivals that mostly died earlier from the metastases in the peritoneal cavity. It can be proposed that targeted treatments in collaboration with reactivated immune system successfully eliminated the micro-metastatic lesions initiated following intracardiac injection of the tumor cells. However, some persistent tumor cells, even if remained undetected during the surgery, may continue to circulate and gain mutations that are favorable for tumor progression. Such mutations may lead to enhanced invasiveness, and eventually, induce penetration to the dynamically changing BBB during disease progression 87 resulting in brain metastases formation. It should be further investigated if a longer continuous combined treatment dosing schedule would be able to eliminate metastasis formation thus prolonging mice survival while improving their quality of life.

Recent clinical trials that incorporate targeted therapies and immunotherapies, show low tolerability to different regimens or combinations. For example, the NCT01400451 trial that evaluated the combination of vemurafenib (BRAFi) and ipilimumab (αCTLA-4) was closed to enrollment after Phase I because the drug combination was not well tolerated 88. Other recent active clinical trials that combine BRAFi/MEKi with immune modulators, such as NCT02967692^37^ and NCT02858921^89^, are monitoring the safety, dose-limiting toxicity, immune microenvironment and biomarker modulations and efficacy. Close attention should be given to the enhanced efficacy together with safety and tolerability of the treatment to the patient. Importantly, when we analyzed the blood chemistry, followed-up weight change and assessed the motor ability by a behavioral study, no toxic effects were shown for the treatment regimen that we used, suggesting that this combination will potentially be better tolerated than those tested clinically. As our *in vivo* model we chose D4M.3A murine melanoma cell line as it bears the BRAF V600E mutation and lack of melanin, which provides the black color of the tumor, as in the case of the B16-F10 melanoma model. We avoided the use of melanin-producing cell lines to visualize the tumor boundaries according to our PCbhQ Turn-ON probe signal and not by a clear black tumor *versus* bright healthy tissue. In addition, the highly activated CTSB in this cell line and tumor tissue was validated by fluorescently-labeled cathepsins ABP, GB123 46, 90, 91. Although D4M.3A cells were considered relatively non-immunogenic, which can be a limitation for immune system-related research, a dramatic increase in immunogenic markers was presented in response to BRAF V600E inhibition 92. Nonetheless, this might explain the relative unresponsiveness of the tumors to ICPi treatment when it was given alone in comparison to its beneficial effect in combination with BRAFi/MEKi. We can speculate that an alternative more immunogenic melanoma model might demonstrate even higher superiority for the addition of immune checkpoint blockade to our PGA-SLM-mDBF treatment. Taken together, our findings may contribute in the future to a more precise, efficacious and safe therapy for preventing melanoma brain metastases.

## Materials and Methods

### Materials

All chemical reagents, including salts and solvents, were purchased from Sigma-Aldrich (Rehovot, Israel) or Tzamal-Dchem (Petach-Tikva, Israel). Cy5-NH_2_ dye was synthesized in our lab by a procedure provided by Prof. Shabat's lab (Tel Aviv University, Israel). SLM was purchased from Petrus-chemicals (Herzliya, Israel) in a minimum of 99% purity. DBF was purchased from Tzamal-Dchem (Petach-Tikva, Israel) in a minimum of 98% purity. All reactions requiring anhydrous conditions were performed under an Ar_(g)_ atmosphere. Chemicals and solvents were either AR grade or purified by standard techniques). Cathepsin B (CTSB) enzyme from the bovine spleen and CA074 me CTSB inhibitor were purchased from Sigma-Aldrich (Rehovot, Israel).

### Cell culture

D4M.3A murine melanoma cells were a kind gift from David Mullins's laboratory (Dartmouth College, Hanover) 92. Advanced DMEM/F12 medium was purchased from Rhenium (Modiin, Israel) and supplemented with 5% Fetal bovine serum (FBS), glutaMAX x100 (Gibco Life Technologies (Carlsbad CA, USA)) and 100 µg/mL streptomycin, 100 IU/mL penicillin, 12.5 IU/mL nystatin. Mouse fibroblasts NIH/3T3 cells were purchased from the ATCC. DMEM medium was purchased from Biological Industries (Kibbutz Beit-HaEmek, Israel) and supplemented with 10% FBS, 100 µg/mL streptomycin, 100 IU/mL penicillin, 12.5 IU/mL nystatin and 2 mM L-Glutamine (Biological Industries (Kibbutz Beit-HaEmek, Israel)). Dulbecco's Phosphate Buffered Saline (DPBS) ×10 solution was purchased from Biological Industries (Kibbutz Beit-HaEmek, Israel). Basal astrocyte medium (AM), astrocytes growth supplement (AGS), endothelial cell growth supplement (ECGS) and 1% penicillin/streptomycin solution were purchased from ScienCell (Carlsbad CA, USA). EndoGRO MV complete medium was purchased from Merck (Germany). The materials for murine endothelial cells (EC) and astrocytes isolation were as following: Collagenase/Dispase solution (Worthington, Lakewood NJ, USA), Red blood cells (RBC) lysis buffer (BioLegend, San-Diego CA, USA) and Percoll (Sigma-Aldrich, Rehovot, Israel). CD31 microbeads for EC separation was purchase from Miltenyi Biotec (Bergisch Gladbach, Germany). Cells were routinely tested for mycoplasma contamination with a mycoplasma detection kit for routinely testing of mycoplasma contamination in cells that was purchased in Biological Industries (Kibbutz Beit-HaEmek, Israel).

### Synthesis of PCbhQ Turn-ON nanoprobe

Poly-L-glutamic acid (PGA) nanocarrier was synthesized as previously described 43, from O-benzyl protected glutamic acid (H-Glu(OBzl)-OH) by NCA polymerization. Starting PGA had an Mw of 15 kD and Đ of 1.024, as characterized by multi-angle static light scattering (MALS). Sulphonated Cy5-NHBoc fluorophore was synthesized by a procedure provided by Prof. Shabat (Tel Aviv University, Israel) 8. The azido derivative of Black Hole Quencher (BHQ-3) was synthesized in our lab according to the procedure previously described 93. First, sulfonated Cy5 was conjugated directly to the PGA backbone (Figure 1A). To activate the fluorophore, the tert-butyloxycarbonyl protecting group (Boc) was removed from the reactive amine by mixing Cy5-NHBoc (15.5 mg, 0.0194 mmol) with trifluoroacetic acid (TFA) and dichloromethane (DCM) mixture (1:1) for 5 min followed by the immediate high-vacuum evaporation. Next, PGA (25 mg, 0.194 mmol) was dissolved in 2 mL anhydrous N,N-dimethylformamide (DMF) and then transferred into the flask containing Cy5-NH_2_. Bis (2-oxo-3- oxazolidinyl) phosphinic chloride (BOP-Cl) (9.867 mg, 0.0388 mmol) was dissolved in 500 µL anhydrous DMF, and 4-dimethylaminopyridine (DMAP) (5.681 mg, 0.0465 mmol) dissolved in 500 µL anhydrous DMF were added to the reaction mixture in order to activate the carboxylic acid residues on the PGA. Finally N,N-diisopropylethylamine (DIPEA) (5 µL, 0.0155 mmol) was added. The reaction was mixed for 4 h in an ice bath and for an additional 48 h at room temperature (RT) Ar_(g)_ atmosphere. The reaction progress was followed by thin-layer chromatography (TLC). At the end, 3 mL of 10% sodium chloride solution (NaCl) was added and the mixture was acidified to pH 2.5 by the addition of 0.5 M hydrochloric acid (HCl) solution. The reaction was stirred for 1 h at RT. The obtained solid was washed three times with acetone/chloroform (4:1) mixture, salted with 0.25 M sodium bicarbonate solution (NaHCO_3_) to make it water-soluble and dialyzed for 48 h at 4°C (Molecular weight cutoff (MwCO) 3.5 kDa) against DI water. Final purification of the product was accomplished by size exclusion chromatography (SEC) using an ÄKTA FPLC system (Amersham Biosciences). The conjugate was dried by freeze-drying and characterized by HPLC (UltiMate® 3000 Nano LC systems, Dionex).

Further, BHQ3-N_3_ was coupled with the obtained PC conjugate using copper-catalyzed azide-alkyne cycloaddition (CuAAC) reaction (click chemistry). To that end PGA-Cy5 (15.5 mg, 0.120 mmol) was dissolved in DMF (1 mL), then propargylamine (0.77 µL, 0.012 mmol) was added. Coupling reagent BOP-Cl (12.22 mg, 0.048 mmol) together with DMAP (15.07 mg, 0.117 mmol) and DIPEA (5 µL, 0.03 mmol) were dissolved in DMF (700 µL) and slowly added to the reaction mixture. The reaction was cooled in an ice bath and stirred overnight under Ar_(g)_ atmosphere. The reaction progress was followed by HPLC (UltiMate® 3000 Nano LC systems, Dionex). At the end, 3 mL of 10% NaCl solution was added and the mixture was acidified to pH 2.5 by addition of 0.5 M HCl solution. The reaction was stirred for 1 h at RT. The precipitate was washed 3 times in DDW and dried by freeze-drying.

Next, BHQ-N_3_ (3.9 mg, 0.00698 mmol) was dissolved in 1000 µL DMSO and added to the PGA-Cy5-propargyl obtained in the previous step. Mix of CuSO_3_ × 5H_2_O (1.04 mg, 0.0029 mmol) and NaAscorbate (5.63 mg, 0.029 mmol) dissolved in 2000 µL DDW and added to the reaction. The mixture was stirred O.N. at RT. Finally, the obtained product was salted with 0.25 M NaHCO_3_ solution to make it water-soluble and dialyzed for 48 h at 4°C (MwCO 3.5 kDa) against DI water.

The loading of the conjugated fluorophore and quencher molecules was determined using SpectraMax M5^e^ multi-detection reader, by means of absorbance, against free Cy5 calibration curve and confirmed by H^1^ NMR (Bruker Avance I and Avance III (Bruker MA, USA) 400 MHz (^1^H)) peak integration. The quenching efficiency of the conjugates was expressed as a percentage of the fluorescence intensity of the conjugates (λ_em_ = 670 nm) compared with the emission of the free Cy5 at the equivalent concentration. Fluorescence spectra measurements were performed using SpectraMax® M5e plate reader or Varioskan LUX Multimode Microplate Reader at λ_ex_/λ_em_ = 630/670 nm. The theoretical Mw of the conjugate was determined according to PGA polymeric precursor Mw, Cy5 and BHQ molecules loading. H^1^ NMR and HPLC characterization is presented (Figure S1).

### Nuclear Magnetic Resonance (NMR)

NMR spectra were recorded using Bruker Avance s Avance I and Avance III (Bruker MA, USA) operated at 400 MHz (^1^H) and 376 MHz (^19^F) spectrometers. Chemical shifts were reported in ppm on the δ scale relative to a residual solvent. Data were analyzed by MestReNova LITE software.

### Dynamic light scattering (DLS) analysis

The mean hydrodynamic diameter determination of PCbhQ conjugate was performed using a Möbius instrument, at 540 nm l.aser wavelength, equipped with a 532 nm longpass filter (Wyatt Technology Corporation, Santa Barbara, CA 93117 USA). The samples were prepared by dissolving 0.5 mg/mL of polymer conjugates in DDW. All measurements were performed at 25°C.

### High Resolution (HR) Transmission Electron Microscope (TEM)

PCbhQ (1 mg/ml in DDW) was adsorbed on formvar/carbon-coated grids. TEM images were taken using a JOEL™ JEM-2010F FEG-STEM (JEOL Ltd., Tokyo, Japan). Diameters were measured by ImageJ software; the particle distribution is the average of 3 fields of view, 10 particles per field.

### Optical Spectral Quantifications

Absorption and emission spectra were obtained with SpectraMax® M5e multi-detection reader or Varioskan LUX Multimode Microplate Reader. Cy5, and BHQ loading was calculated according to the absorption of the conjugate at 650 or 750 nm compared to the free Cy5 or quencher calibration curves. Quenching efficiency was determined after determining the loading. It was expressed as a percentage of the fluorescence intensity of the quenched conjugates bearing Cy5 (λ_em_ = 670 nm) compared to the emission of the free Cy5, at an equivalent concentration.

### Enzymatic activity assays

#### The degradation of imaging probes with CTSB

To study enzymatically driven degradation of the conjugates followed by fluorescence signal increase *in vitro*, PC and PCbhQ conjugates were incubated with CTSB from the bovine spleen (1 U/mL). The assay was performed at 37 °C, in freshly prepared activity phosphate buffer, pH 5.5, containing 0.1 M sodium phosphate, 0.05 M NaCl, 1 mM Ethylenediaminetetraacetic acid (EDTA) and 5 mM reduced glutathione (GSH). As controls, conjugates were incubated in the absence of CTSB or in the presence of CTSB selective inhibitor, CA-074 Me, (200 µM) at the same conditions or in the physiological conditions (PBS, pH = 7.4). The degradation of the probes was monitored by measuring the fluorescence signal in sequential time points up to 55 h. The fluorescence measurements were carried out using SpectraMax M5^e^ multi-detection reader (λ_ex_/λ_em_ = 630/670 nm, filter: 665 nm). The measured fluorescence signal was normalized to the initial signal, where each measurement (F_x_, i.e, fluorescence at time x) was divided by the fluorescence signal obtained in time zero (F_i_, i.e., initial fluorescence).

#### In vitro cellular uptake, co-localization with lysosomes and fluorescence signal Turn-ON of PCbhQ probe

To evaluate cellular uptake, trafficking and *in vitro* Turn-ON abilities of the imaging probe, 2 x10^6^ D4M.3A murine melanoma cells were seeded onto 10 cm petri dishes. Day after, cells were treated with 250 nM Cy5 eq. concentration of PCbhQ or free Cy5 in the same concentration for 0.5 h. Next, the treatment-containing medium was exchanged to the cell's growth medium in which the cells were incubated for 1, 4 and 24 h. The lysosomes were stained by the addition of 100 nM LysoTracker™ Green DND-26 (Life technologies) half an hour before the completion of each time point incubation time. Finally, cells were washed 3 times with PBS and detached from the petri dish by phenol-red free trypsin (0.5% Trypsin EDTA x10 solution diluted in PBS). Then, 6 mL of 2% FBS/ PBS were added, and the cells were precipitated by 7 min centrifugation at 1100 rpm (4 °C). The cells were resuspended in 50 µL of 2%FBS/PBS and analyzed by ImageStream®^X^ Mark II Imaging Flow Cytometer for cellular uptake, co-localization with lysosomes, or fluorescence Turn-On by fluorescent signal evaluation. The analysis was performed by means of IDEAS® Image data exploration and analysis software. To determine the co-localization degree between Cy5 and LysoTracker™ Green DND-26, similarity feature, which is a log transformed Pearson's Correlation Coefficient and is a measure of the degree to which two images are linearly correlated within a masked region was implemented. The Cy5 positive threshold was determined according to untreated cells.

#### 3D spheroids for tumor sprouting evaluation

3D tumor spheroids of primary and brain metastatic melanoma were prepared using the hanging-drop method as reported before 94. The spheroids were generated by co-culturing melanoma D4M.3A mCherry-labeled cells with either skin (freshly isolated unlabeled murine dermal fibroblasts, DF) in 1:1 cell ratio or brain (unlabeled astrocytes, endothelial cells, microglia and pericytes) stromal cells in 1:1:2:0.5:0.5 cell ratio. In both cases the total number of cells was 80,000 cell/mL. The spheroids grown in reduced growth factor Matrigel® (BD, Franklin Lakes NJ, USA) together with splenocytes activated with αCD28 and rhIL-2 (1:75, tumor cells to splenocytes). The co-cultures (3D spheroids and splenocytes) were treated with mDBF and SLM drugs, free or conjugated to PGA nanocarrier (1.75 µM mDBF, 3 µM SLM) alone or in combination with ICPi, monoclonal antibodies (αPD1, αCTLA-4) or with ICPi alone. As a control, spheroids were treated either with PBS or PGA nanocarrier. Melanoma cell sprouting was monitored for 24 h. and visualized using EVOS FL Auto cell imaging system (Thermo Fisher Scientific, Waltham MA, USA).

### Animals and Tumor Cell Inoculation

#### The randomization and assignment of mice to the treatment groups

In order to avoid bias in the results, two randomization steps were implemented during the study. First, mice were randomly divided into 2 groups (IGS/non-IGS) for the tumor excision step. Afterwards, mice from both groups underwent the procedure of the metastasis induction. Before starting the antimetastatic treatments second randomization step was implemented. All mice were randomly assigned to one of the treatment groups.

#### Primary tumor inoculation

Male C57BL/6J mice (aged 6-8 weeks) were inoculated subcutaneously (*s.c.*) with 1x10^6^ in 100 µL saline D4M.3A murine melanoma cells at a dorsal site (above the kidneys line). Before inoculation, the mice were anesthetized with 100 mg/kg Ketamine and 10 mg/kg Xylazine and shaved at the injection area. Mice were monitored every other day for body weight changes and tumor growth was measured using a caliper. Tumor volume was calculated using the standard formula: length × width^2^ × 0.52. Tumor-bearing mice that did not undergo surgery or treatment were euthanized 22 days post inoculation according to IACUC regulations (tumor volumes were above 1000 mm^3^).

#### Intracardiac Metastases Induction

D4M.3A-Luc (luciferase-labeled) cells (1x10^6^ cells in 100 µL saline) were inoculated into the left ventricle of C57BL/6J mice, under ultrasound (US) supervision, 7 days post primary tumor resection. Before the procedure animals were anesthetized with 100 mg/kg Ketamine and 10 mg/kg Xylazine and the fur was removed from the injection area by means of shaving.

### Imaging Techniques

#### Fluorescence Optical Imaging (FLI)

Fluorescence signal emerged within the primary tumor or metastatic lesions following fluorescent molecules injection to the mice was assessed using non-invasive imaging system CRI Maestro^TM^. Multispectral image-cubes were acquired through 650-800 nm spectral range in 10 nm steps using excitation (λ_ex_ = 635 nm longpass) and emission (λ_em_ = 675 nm longpass) filter set. Mice auto-fluorescence and undesired background signals were eliminated by spectral analysis and linear unmixing algorithm.

#### Bioluminescence Optical Imaging (BLI)

To monitor the development of the metastatic lesions over time, mice that were inoculated i.c. with D4M.3A-LUC melanoma cells were imaged by IVIS® Lumina III *in vivo* Imaging System three times a week. D-Luciferin bioluminescent reporter (150 µL of 7.5 mg/mL dissolved in DPBS) was injected intraperitoneal (*i.p.*) Mice were euthanized with 100 mg/kg Ketamine and 10 mg/kg Xylazine 10 min. post the substrate injection and have been imaged bilaterally. Exposure time was set to 5 min each side.

#### Magnetic Resonance Imaging (MRI)

Brain metastases development were confirmed by 4.7T MRI - MRS 4000™ (MR solutions). Mice were anesthetized by Ketamine (100 mg/kg) and Xylazine (10 mg/kg) injected *i.p.* and T1- weighted with contrast agent (Magnetol, Gd-DTPA, Soreq M.R.C. Israel Radiopharmaceuticals) images were obtained. The time scan was set to 5 min.

#### PCbhQ probe pharmacokinetics (PK) study

PCbhQ Turn-ON probe was injected *i.v.* to C57BL/6J male mice bearing D4M.3A murine melanoma tumors. At predetermined intervals of 0, 15, 30 and 60 min, blood samples (200 μL) were taken from the submandibular vein and kept on ice. The samples were then centrifuged at 350 rcf for 10 min and 50 µL plasma supernatant samples were collected. The fluorescence signal of the Cy5 bearing conjugates inside the plasma was evaluated by SpectraMax M5e multi-detection reader at λex/λem = 630/670 nm.

#### PCbhQ probe biodistribution and tumor accumulation

Male C57BL/6 mice were inoculated subcutaneously (*s.c.*) with 1 × 10^6^ D4M.3A murine melanoma cells. When tumors reached an average volume of 150 mm^3^, PCbhQ Turn-ON probe was injected to mice *i.v.* (40 µM Cy5-eq. concentration, 200 µL PBS). Tumor accumulation was monitored using CRI Maestro^TM^ imaging system at several predetermined timepoints (15 and 30 min.,1, 3.5, 7 and 24 hours). Before imaging mice were anesthetized using ketamine (100 mg/kg) and xylazine (12 mg/kg) and shaved. After 3.5 h and 24 h group of mice were euthanized, and organs and tumor were imaged ex-vivo. Multispectral image-cubes were acquired through 590-750 nm spectral range in 10 nm steps using excitation (635 nm) and emission (675 nm) filter set. Mice auto-fluorescence and undesired background signals were eliminated by spectral analysis and the Maestro linear unmixing algorithm.

#### Image-Guided Surgery of the primary tumors

Mice bearing D4M.3A tumors were randomly divided into two groups. The first group was assigned for image-guided tumor resection (IGS) under fluorescence guidance of PCbhQ (n = 31 mice), while the second group underwent surgery under white light (n = 35). Animals were anesthetized with 100 mg/kg Ketamine and 10 mg/kg Xylazine and fur was removed from the tumor area by shaving. All surgical procedures were performed in sterile conditions. The IGS group was injected *i.v.* with a PCbhQ probe (40 µM Cy5-eq. concentration, 200 µL saline). The time point for surgery beginning was chosen to be 3.5 h post *i.v.* injection according to the fluorescent signal kinetics evaluations. Intravital imaging by CRI Maestro^TM^ Fluorescence Imaging System was performed as described above, during all surgery steps. Images of the fluorescence signal were displayed on an adjacent monitor and all Cy5-positive tissue foci (suspected as tumor tissues) were excised. The control groups underwent surgery under white light illumination. Hemostasis was achieved with a handheld hemostat. Following completion of tumor removal, which was assessed by fluorescent signal (background level) or white light (control group), skin incisions were sewed, and animals were returned to their cages to recover from the anesthesia.

### Evaluation of primary tumor growth inhibition by PGA-SLM-mDBF alone or in combination with ICPi

Starting from 10 days following primary tumor inoculation (~ 150 mm^3^ tumor volume) mice, N=5 per group were treated with DBF/SLM combination at 10 and 15 mg/kg free drugs-equivalent dose, respectively. The treatments were administered every day (Q.D.) i.p., as a cocktail of free drugs or PGA-conjugated to both drugs. Four days post BRAFi/MEKi treatments initiation, ICPi (αPD-1 and αCTLA-4, 5 mg/kg each), administered twice a week (B.I.W.) were added to the therapeutic dosing schedule of two treatment groups. The control groups were treated with PBS or vehicle (0.1:1:1:8 DMSO:Chremophor:EtOH:saline). The PGA-SLM-mDBF was dissolved in PBS, while free drugs required the vehicle described above as they are not water-soluble. Tumor volume was measured by a digital caliper using width × 2 × length × 0.52 formula for tumor volume calculation. In addition, body weight was monitored. Mice were euthanized if tumor volume reached 1000 mm^3^ or if they lost more than 15% of their body weight. The tumor growth curves were stopped twenty-two days post tumor inoculation. Safety profile and molecular mechanism of free and PGA-conjugated drugs were evaluated following the termination of the treatments.

### Evaluation of the safety profile of free and PGA-conjugated BRAFi/MEKi

#### Blood count and chemistry

To test the effect of treatments in vivo on mice blood chemistry and blood count, blood was collected from submandibular vein of mice 24 hours following last treatment administration. Blood samples were analyzed by AML Ltd (Herzliya, Isreal).

#### Motor coordination test

DBF- and SLM- related motor coordination was assessed by a Rotarod apparatus (Columbus Instruments, OH, USA). Mice were tested following the last treatment with 10 mg/kg DBF and 15 mg/kg SLM, as free or PGA-conjugated, respectively. The control groups were mice treated with PBS or vehicle (0.1:1:1:8 DMSO:Chremophor:EtOH:saline). The initial speed was 1.6 rpm, with an acceleration rate of 4 rpm per min. Animals were tested five times during each session, with at least 2 min. of rest between each test. The three best performances of each mouse were considered, and the results were averaged for the whole group.

#### Tissue Histology

Excised tissues, both following IGS or BRAFi/MEKi treatments (22 days post tumor inoculation) were processed and OCT/paraffin-embedded for further histopathological analysis. For OCT embedding, excised tissues were incubated with 4% PFA for 3 h followed by 0.5 M sucrose (BioLab) solution for 1 h and 1 M sucrose O.N. For paraffin-embedded fixation, tissues were kept 48 h in 4% PFA, and then applied in Tissue-Processor (Leica) for 10 h and embedded in paraffin using Leica Tissue Embedder. Prior to histopathological evaluation 8 µm sections were obtained.

Immunostaining of the frozen sections was performed using the BOND RX automated IHC stainer (Leica Biosystems, Wetzlar, Germany). Briefly, slides were incubated with goat serum (10% goat serum in PBS X1 + 0.02% Tween-20) for 30 min to block nonspecific binding sites. Slides were then incubated with rabbit anti-mouse Ki67 (1:50 dilution, Novus biologicals, Centennial, CO, USA), rabbit anti-mouse cleaved caspase 3 (1:30 dilution, Cell Signaling, Danvers, MA, USA), rabbit anti-mouse phospho-BRAF (1:100 dilution, Novus biologicals, Centennial, CO, USA, rabbit anti-mouse phospho-ERK (1:30 dilution, Novus biologicals, Centennial, CO, USA), rat anti-mouse CD8 (1:50 dilution, BioLegend, San Diego, CA, USA), rabbit anti-mouse PD-1 (1:100 dilution, Proteintech, Rosemont, IL, USA), rat anti-mouse CD4 (1:100 dilution, BioLegend, San Diego, CA, USA) and rabbit anti-mouse FOXP3 (1:100 dilution, Novus biologicals, Centennial, CO, USA). After 1 h incubation, slides were incubated for an additional 1 h with the secondary antibody goat anti-rabbit Alexa-647 (1:350 dilution, Abcam, Cambridge, UK) or goat anti-rat Alexa-488 (1:350 dilution, Abcam, Cambridge, UK) followed by Hoechst fluorescent dye (1:5000 dilution, ThermoFisher Scientific, Inchinnan Business Park Paisley PA4 9RF, UK) for an additional 10 min for nuclei counterstaining. The tissues stained were then fixed and mounted on a glass microscope slide with a glass coverslip using ProLong Gold antifade reagent (Invitrogen). Fluorescence images were captured using a fluorescence microscope (Evos FL Auto, Life Technologies) at 40-x magnification.

### Evaluation of PGA-SLM-mDBF capacity in the prevention of metastases development and primary tumor recurrence, alone or in combination with ICPi

Seven days post the resection of the tumors, the metastases were induced (i.c.) to mice from both IGS and non-IGS groups. Before starting the treatments second randomization step was implemented. All mice were randomly assigned to one of the treatment groups. The preventive, antimetastatic treatments were initiated four days following i.c. injection of D4M.3A-LUC cells. DBF/SLM (n = 8), PGA -SLM-mDBF (n = 8), DBF/SLM+ICPi (n = 9), PGA-SLM-mDBF +ICPi (n = 10) and ICPi (n = 10) alone were injected to mice *i.p.* The drugs (BRAFi/MEKi, 10/15 mg/kg), free or conjugated were administered every day (Q.D.) for 14 days. Immune checkpoint inhibitors (αPD-1 and αCTLA-4, 5 mg/kg each) were added twice a week (B.I.W.). The control groups were treated with PBS or vehicle (0.1:1:1:8 DMSO:Chremophor:EtOH:salin) (n = 7 per each group). The PGA-SLM-mDBF was dissolved in PBS, while free drugs required the vehicle described above. Metastasis development was monitored three times a week by bioluminescence signal (BLI) assessed utilizing IVIS® Lumina III *in vivo* Imaging System (PerkinElmer). Brain metastases was confirmed by MRI. Solid tumors were measured by a digital caliper and tumor volume was calculated as width^2^ × length × 0.52. In addition, body weight was monitored. Mice were euthanized when tumor volume reached 1000 mm^3^ or when they lost more than 15% of their body weight. For the metastases development assessment, we included only mice with confirmed metastatic spread.

### Metastasis identification by optical fluorescence signal assisted by PCbhQ Turn-ON probe

Mice reached one of the predetermined exclusion criteria, such as weight loss (more than 10% or 15% from the subsequent or reference weighting, respectively), tumor recurrence size above 400 mm^3^ and/or neurological disorders were assessed by CRI Maestro^TM^ imaging for metastatic spread and then euthanized. PCbhQ Turn-ON probe was injected *i.v* (40 µM in 200 µl PBS) 3.5 h before imaging. Metastatic lesions were validated histologically by H&E staining.

### Statistical Methods

Normalization, if performed, was noted at the relevant figure. All in vitro studies data are presented as mean ± SD, while the data from the in vivo studies are presented as mean ± SEM. Sample size (n) for each statistical analysis was added for each experiment. Statistical significance was determined using two-sided Student's t-test, one-way ANOVA, or two-sided repeated-measures ANOVA with P-value testing level. Kaplan-Meier curve was created to assess the survival of mice *in vivo*. The software used for statistical analysis was GraphPad Prism 8.

### Ethics Statement

All animal procedures were approved and performed in compliance with the standards of Tel Aviv University, Sackler School of Medicine Institutional Animal Care and Use Committee (IACUC).

## Supplementary Material

Supplementary materials and methods, and figures.
