# Supplementary material for: Polyglutamate-based nanoconjugates for image-guided surgery and post-operative melanoma metastases prevention
Source: Theranostics. 2022 Aug 29;12(14):6339–62. doi: 10.7150/thno.72941 (PMC9475454; doi:10.7150/thno.72941)
Supplement: Supplementary file 1 — Supplementary materials and methods, and figures. [file thnov12p6339s1.pdf]

Supplementary Material

# Polyglutamate-based nanoconjugates for image-guided surgery and post-operative melanoma metastases prevention


Yana Epshtein[1†], Rachel Blau[1,2†], Evgeni Pisarevsky[1†], Shani Koshrovski-Michael[1], Dikla Ben-Shushan[1], Sabina Pozzi[1], Gal Shenbach-Koltin[1], Lidar Fridrich[1], Marina Buzhor[1], Adva Krivitsky[1], Pradip Dey[1], and Ronit Satchi-Fainaro[1]*

*[1]Department of Physiology and Pharmacology, Sackler Faculty of Medicine, Tel Aviv University, Tel Aviv 6997801, Israel.*

*[2]Department of NanoEngineering, University of California, San Diego, 9500 Gilman Drive, Mail Code 0448, La Jolla, CA 92093-0448*

† These authors contributed equally to the work

* Author to whom correspondence should be addressed:

Prof. Ronit Satchi-Fainaro, Ph.D.

Department of Physiology and Pharmacology

Sackler Faculty of Medicine, Room 607

Tel Aviv University, Tel Aviv 6997801, Israel

Tel: 972-3-640-7427

E-Mail: ronitsf@tauex.tau.ac.il


This file includes:

Supplementary Material and Methods

Supplementary Figures

**Nuclear Magnetic Resonance (NMR)**

NMR spectra were recorded using Bruker Avance s Avance I and Avance III (Bruker MA, USA) operated at 400 MHz ($^{1}$H) and 376 MHz ($^{19}$F) spectrometers. Chemical shifts were reported in ppm on the δ scale relative to a residual solvent. Data were analyzed by MestReNova LITE software.

**High-Pressure Liquide Chromatography (HPLC)**

Analytical HPLC was used for the characterization of the various substances. UltiMate® 3000 Nano LC systems (Dionex) was equipped with 3000 pump, VWD-3000 UV-Vis, fluorescence detector and Chromeleon® 6.80 software. The columns in use are Waters 3.9 X 150 mm C-18 RP (reversed-phase) and Phenomenex Jupiter 5 µm 250 X 4.60 mm C-18 300A. Chromatographic conditions: flow: 1.0 mL/min, gradient: 20% to 100% solution B in 20 min (solution A - 0.1% TFA in water; solution B - 0.1% TFA in acetonitrile (ACN)).

**NIH-3T3 cell viability assay**

NIH-3T3 cells were plated on 96-well plates (1000 cells/well) in 100 µL DMEM medium and allowed to attach for 24 h. Cells were then incubated with several concentrations of PCbhQ (up to 50 µM Cy5-eq concentration), dissolved in the appropriate cell culture medium, for 24 h. Following incubation, 50 µL XTT solution were added to each well and plate was incubated for another 4 h. Triton was used as positive control. To assess cell viability, spectrophotometrical absorbance was measured using spectramaxELISA microplate (ELISA) reader (λ=450 nm).

**In vitro inhibition of CTSB activity**

To determine the CA-074 Me, inhibitor concentration suitable for the inhibition of the ability of 1 U/mL CTSB to activate the Turn-ON probe (10 µM Cy5 eq.), the concentration dependence assay was performed. To that end, 0.2 U/mL of CTSB was challenged with different inhibitor concentrations (0-250 µM). The inhibition of CTSB activity was evaluated by measuring the fluorescence signal intensity emitted by enzymatically degraded conjugate at sequential time points. The fluorescence measurements were carried out at an excitation wavelength of 630 nm using SpectraMax® M5e multi-detection reader. The fluorescence images were acquired with CRI Maestro™ (λex/λem = 635/675 nm).

**Synthesis of PGA-SLM-mDBF therapeutic conjugate**

PGA-SLM-mDBF conjugate synthesis was designed and evaluated in our lab as described in Pisarevsky *et al*. [1]. Briefly, prior to conjugation DBF was modified with a diol functional group to become DBF-diol (mDBF). PGA (800 mg, 6.2 mmol monomers, Mw = 10 kDa, Đ = 1.16), SLM (0.93 mmol, 425 mg) and mDBF (0.31 mmol, 183 mg) were dissolved in 40 mL anhydrous DMF and cooled in an ice-bath for 15 min. BOP-Cl (2.5 mmol, 635 mg), DMAP (5 mmol, 610 mg) and DIPEA (252 µL, 1.56 mmol) were added and stirred for 4 h in an ice-bath, and then heated to RT overnight. Following the hydrolysis that was performed by 10% NaCl and 0.2 mL, 0.5 M HCl the product was washed 3 times with DDW and freeze-dried to powder. The elimination of the unconjugated drug was reached through repeating washes with ACN following 48 h dialysis (MWCO=3.5 kDa) against DDW. The drug loading was measured by $^{19}F$ NMR by addition of 0.002 mmol of the internal standard $CF_3CH_2OH$ to 5 mg of the conjugate and peaks area calculation.

**Multi-angle static light scattering (MALS)**

MW and Đ analysis of PGA polymer were performed on Agilent 1200 series HPLC system (Agilent Technologies Santa Clara, CA, US) equipped with a multi-angle light scattering detector (Wyatt Technology Corporation Santa Barbara, US), using Shodex Kw404-4F column (Showa Denko America, Inc.) in PBS, flow 0.3 ml/min.

**Fast Protein Liquid Chromatography (FPLC)**

FPLC (ÄKTA, Amersham Biosciences) equipped with a UV detector was used for the preparative separation of various substances. Polymers were separated from small molecules by SEC using HiTrap desalting columns (Sephadex G-25 Superfine) in DDW with a flow rate of 1.0 mL/min; UV/Vis detection was monitored at 220, 600 and 650 nm.

**Magnetic Resonance Imaging (MRI)**

Brain metastases development were confirmed by 4.7T MRI - MRS 4000™ (MR solutions). Mice were anesthetized by Ketamine (100 mg/kg) and Xylazine (10 mg/kg) injected *i.p.* and T1-weighted with contrast agent (Magnetol, Gd-DTPA, Soreq M.R.C. Israel Radiopharmaceuticals) images were obtained. The time scan was set to 5 min.

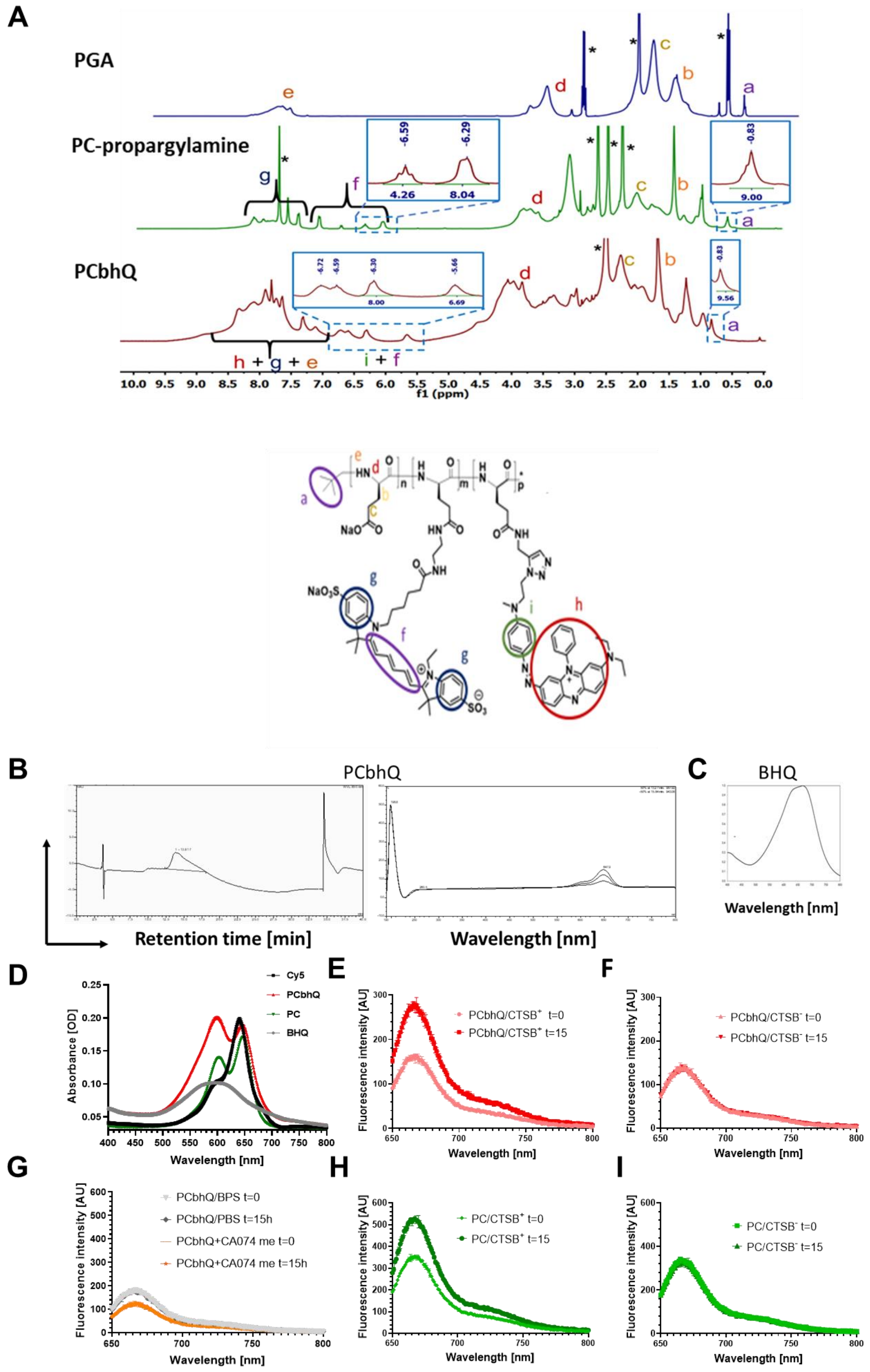


**Figure S1: Characterizations of PCbhQ (A)** $^{1}$H NMR spectrum of PCbhQ synthesis steps. Upper panel: PGA, Middle panel: propargylamine conjugation step, Lower panel: Final PCbhQ product.

The main peaks are assigned to the corresponding protons of the structure drawn below the spectra. (**B-C)** HPLC spectrum and chromatogram of PCbhQ Turn-ON probe and of the unconjugated BHQ molecule. **(B)** Left: PCbhQ conjugate chromatogram. Right: corresponding spectrum, with broad peak at $\lambda_{max}$ = 670 nm, indicating BHQ presence (Cy5 corresponding peak is not shown as it undercovered by BHQ peak). **(C**) Representative spectra of unconjugated BHQ ($\lambda_{max}$ = 670 nm) molecule. HPLC running conditions: gradient 20-100% ACN:DDW with 0.01% TFA, flow rate: 1 mL/min, room temperature. **(D)** UV-vis spectra of PCbhQ, PC, free Cy5 and BHQ measured in PBS. **(E-I)** Fluorescence spectra of PCbhQ and PC measured during incubation of the probes in the presence (E and H) and in the absence (F and I) of CTSB measured at the t=0 and following 15 hours of incubation. In addition, PCbhQ spectra were measured in PBS and in the presence of CTSB and CA074 me CTSB inhibitor (G). All measurements were performed using Varioskan LUX Multimode Microplate Reader. Data represent mean (n = 3 technical repeats).

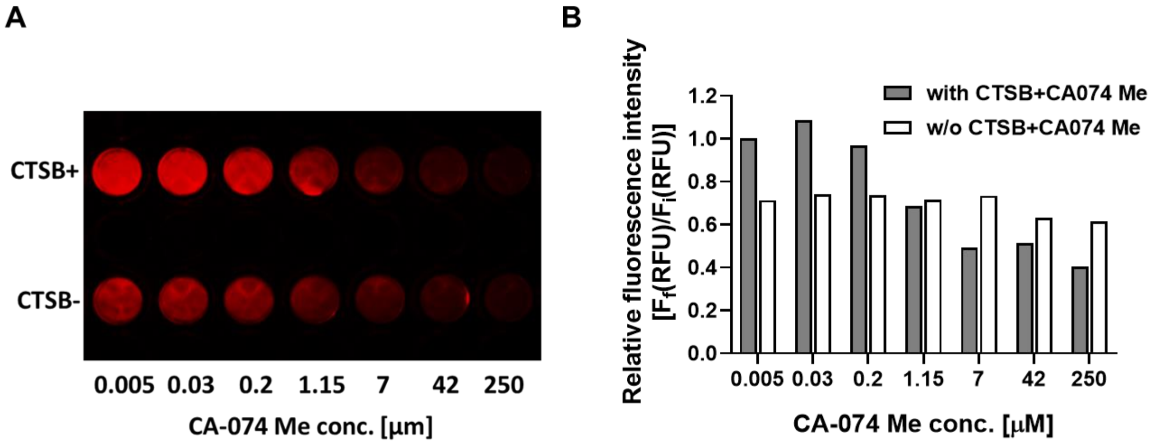


**Figure S2: CTSB inhibitor concentration correlates with CTSB activity inhibition.**

(**A**) The fluorescence signals and **(B)** its quantification obtained 26 h post PCbhQ Turn-ON probe incubation with CTSB inhibitor in the presence or absence of CTSB.

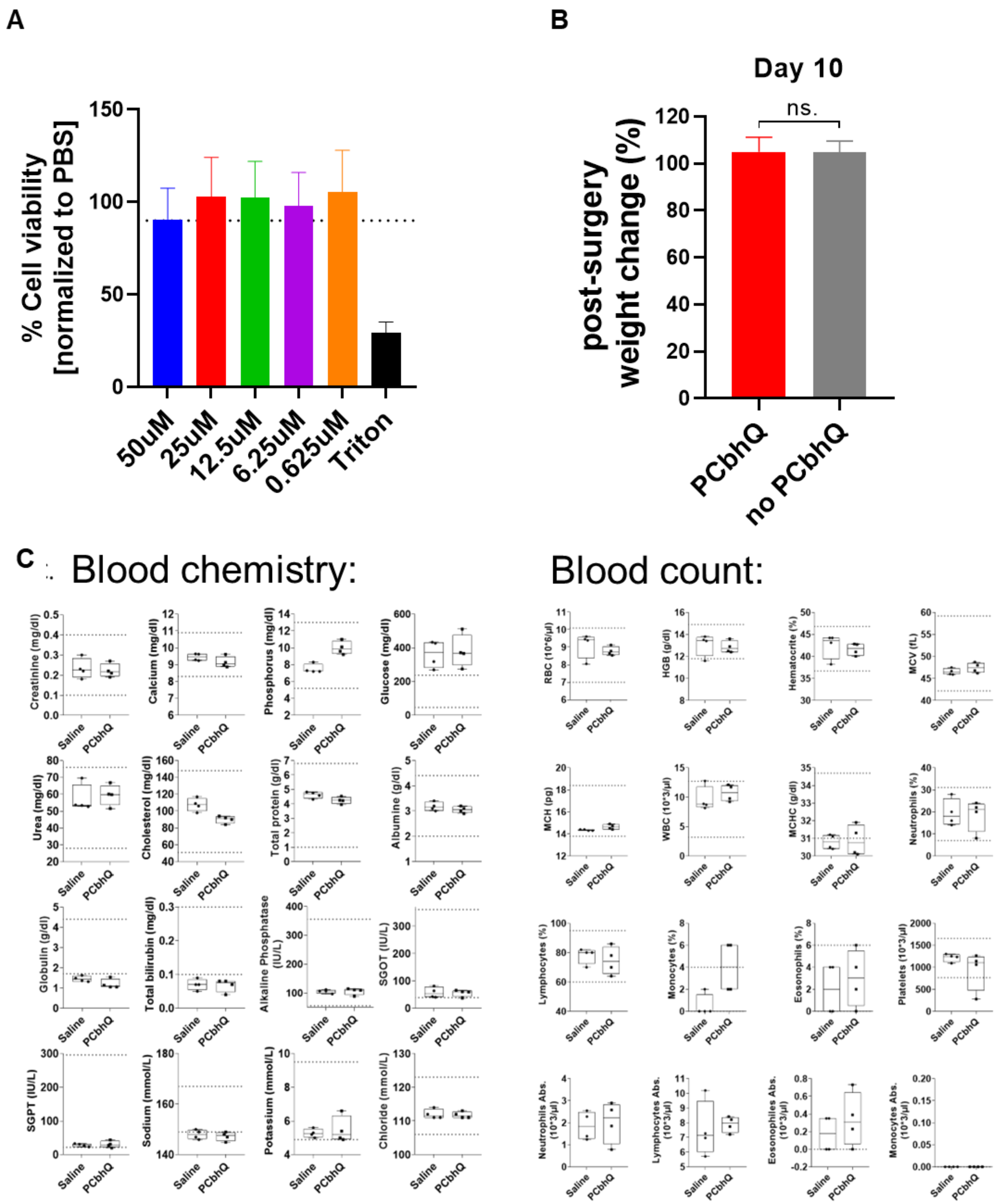


**Figure S3: The evaluation of biotoxicity of PCbhQ. (A)** PCbhQ had no cytotoxic effect on NIH3T3 normal fibroblasts cell viability. **(B)** No weight change was observed following PCbhQ

single administration. **(C)** Blood count and blood chemistry evaluation following PCbhQ single administration.

A

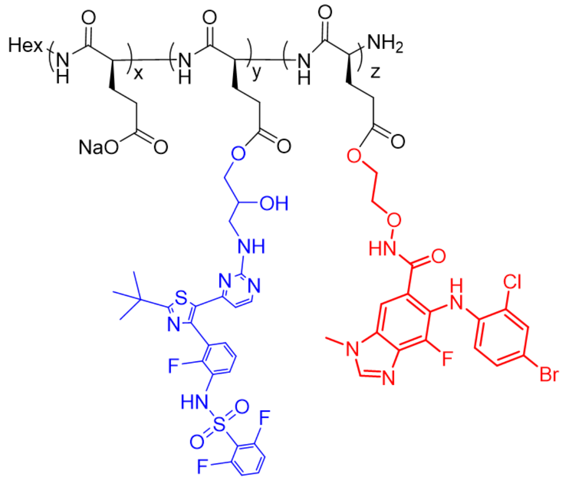


B

| Conjugate | Size by DLS [nm] | Drug loading DBF [wt%] | Drug loading SLM [wt%] | Zeta potential [mV] |
|---|---|---|---|---|
| **PGA-mDBF-SLM** | 11.7±6.7 | 12 | 16 | -35.82 |

**Figure S4: Schematic represenration and characterization of PGA-SLM-mDBF therapeutic conjugate. (A)** Schematic representation of PGA-mDBF-SLM. (**B)** The summary of physico-chemical characterization of PGA-mDBF-SLM. Drug loading was optically calculated using a calibration curve or by $^{19}$F-NMR using internal standard of Trifluoroethanol (-77 ppm).

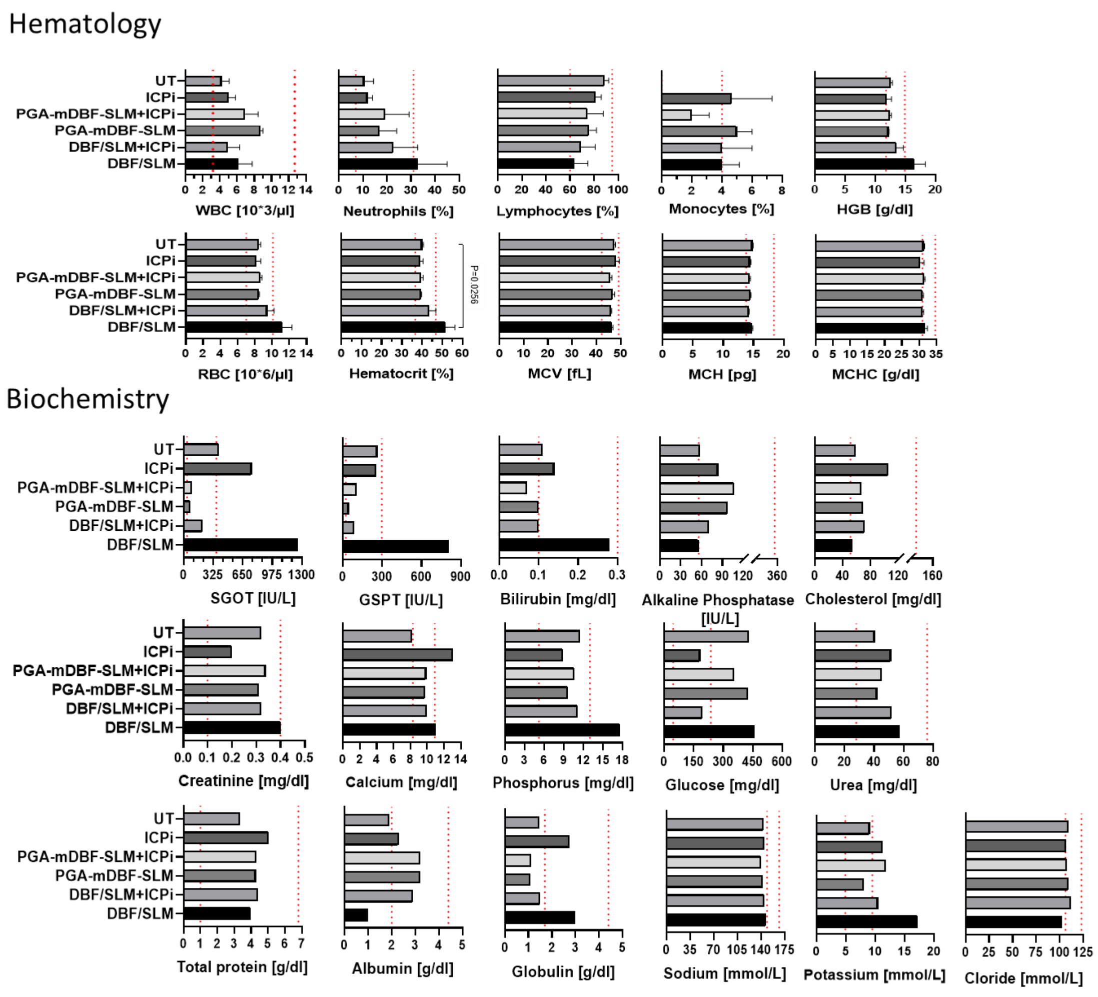


**Figure S5: Evaluation of toxic effects of PGA-mDBF-SLM - blood tests.** Blood samples collected from submandibular vein 24 h post last treatment was evaluated for blood count and biochemistry for possible toxic effects. Mice treated with free drugs demonstrated meaningful increase in kidney and liver function parameters suggesting drug-induced toxicity effect compared to conjugate-treated groups that did not show this increase. N=3 mice per group.

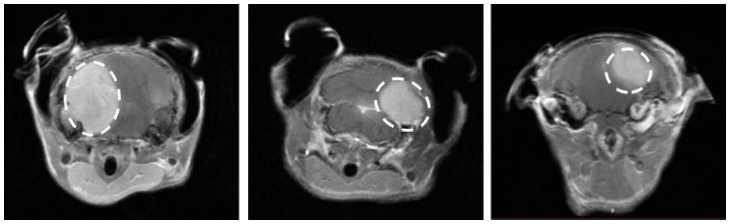

**Figure S6: Magnetic resonance imaging (MRI) of brain metastases.** The representative images of mice imaged by MRI following the neurological symptoms.
